# Antioxidant Activity and Discrimination of Organic Apples (*Malus domestica* Borkh.) Cultivated in the Western Region of Romania: A DPPH· Kinetics–PCA Approach

**DOI:** 10.3390/plants10091957

**Published:** 2021-09-19

**Authors:** Olimpia Alina Iordănescu, Maria Băla, Alina Carmen Iuga, Dina Gligor (Pane), Ionuţ Dascălu, Gabriel Stelian Bujancă, Ioan David, Nicoleta Gabriela Hădărugă, Daniel Ioan Hădărugă

**Affiliations:** 1 Department of Horticulture, Banat’s University of Agricultural Sciences and Veterinary Medicine “King Michael I of Romania” from Timişoara, Calea Aradului 119, 300645 Timişoara, Romania; olimpia.iordanescu@yahoo.com (O.A.I.); mariabalamonicabala@yahoo.com (M.B.); i_ali4ever@yahoo.com (A.C.I.); dascalu_ionut91@yahoo.com (I.D.); 2 Doctoral School “Engineering of Vegetable and Animal Resources”, Banat’s University of Agricultural Sciences and Veterinary Medicine “King Michael I of Romania” from Timişoara, Calea Aradului 119, 300645 Timişoara, Romania; gligor_dina_bihor@yahoo.com (D.G.); daniel.hadaruga@upt.ro (D.I.H.); 3 Department of Food Control, Banat’s University of Agricultural Sciences and Veterinary Medicine “King Michael I of Romania” from Timişoara, Calea Aradului 119, 300645 Timişoara, Romania; gabrielbujanca@yahoo.com; 4 Department of Food Science, Banat’s University of Agricultural Sciences and Veterinary Medicine “King Michael I of Romania” from Timişoara, Calea Aradului 119, 300645 Timişoara, Romania; 5 Department of Applied Chemistry, Organic and Natural Compounds Engineering, Polytechnic University of Timişoara, Carol Telbisz 6, 300001 Timişoara, Romania

**Keywords:** apple, *Malus domestica* Borkh., antioxidant activity, organic and non-organic orchards, DPPH· kinetics, mean DPPH· reaction rate, principal component analysis discrimination

## Abstract

Apple (*Malus domestica* Borkh.) is one of the most used fruit for beverages in Romania. The goal of the study was to evaluate the antioxidant activity and discrimination of various parts of organic and non-organic apple varieties cultivated in the western region of Romania using the DPPH· kinetics–PCA (principal component analysis) approach. Organic and non-organic apples were subjected to solid–liquid ethanol extraction. Core and shell extracts were mixed with DPPH· and spectrophotometrically monitored at 517 nm. Antioxidant activity and mean DPPH· reaction rate at various time ranges reveal significant differences between organic and non-organic samples, as well as apple parts. Organic core and shell extracts had higher antioxidant activities than the corresponding non-organic samples (74.5–96.9% and 61.9–97.2%, respectively, 23.5–94.3% and 59.5–95.5%). Significant differences were observed for the DPPH· reaction rate for the first ½ min, especially in the presence of organic core extracts (3.7–4.8 μM/s). The organic samples were well discriminated by DPPH· kinetics–PCA, the most important variables being the DPPH· reaction rate for the first time range. This is the first DPPH· kinetics–PCA approach applied for discriminating between organic and non-organic fruits and can be useful for evaluating the quality of such type of fruits.

## 1. Introduction

Apples (*Malus domestica* Borkh.) have been known and cultivated since ancient times, both in Asia (Anatolia and Persian Empire) and Europe (Roman Empire) [1]. China is the main producer with more than 42.4 Mt in 2019 and an area harvested of more than 2 million of ha [2]. It is followed by the United States of America, Turkey, Poland and India. On the other hand, New Zealand is on the top of the pile regarding apple yield. Romania has 52740 ha of cultivated area and an apple production in 2019 of 2.316 × 10^6^ tons (the fourteenth and thirtieth positions, respectively) [2]. Nowadays, more than ten thousand of apple cultivars exist. Among these cultivars, “Golden Delicious”, “Florina”, “Generos” and “Starkrimson” are mainly cultivated in Romania [2,3,4]. “Golden Delicious” is a yellow winter apple originating from the United States of America. It is a hybrid of “Grimes golden” and “Golden reinette” cultivars and provides higher productivity and quality [5,6,7,8]. The “Florina” cultivar is a winter apple that comes from France. The color is a mixture of yellow–green, with red stripes. It is a combination of “Jonathan”, “Golden Delicious” and “Rome” varieties [9]. “Generos” variety was developed in Romania in 1985 from “Parmain d’or × M. Kaido”, “Jonathan × V 53-39-2” and “Frumos de Voineşti × V 60-6-51” varieties [10]. It is a winter apple and is resistant against diseases and pests. “Starkrimson” is an apple variety resulting from the “Stark” and “Delicious” cultivars [11,12]. The “Starkrimson” apples are red-colored and have longer shelf life.

Apples are consumed as fresh fruits, but also as other food and beverage products after processing. The quality and chemical composition of apples are changed during storage (cold storage, controlled atmosphere), and especially after processing in order to obtain apple beverages (juice, cider, vinegar, alcoholic beverages) or other food products (jam, sauce, dry or canned apples) [13,14,15,16]. Moreover, by-products from apple processing are also used for obtaining various beverages such as apple tea and filter tea, or biologically active compounds by extraction, designed for functional foods [16,17,18]. Apple processing includes mechanical, thermal and chemical pre-treatments, osmotic dehydration, drying, irradiation, fermentation, clarification, extraction, concentration, steaming, filtration, etc. [8,13,15,16]. The main compounds affected by processing are antioxidants, among other nutrients. Fresh apples have a high content of carbohydrates of about 13.8%, especially sugars such as sucrose, glucose and fructose (a total of 10.4%) [1]. Other constituents are dietary fibers, lipids, proteins, pectins, minerals (K, Ca, Mg, Fe and P), vitamins and carotenoids (vitamins C, A, E, β-carotene, lutein and zeaxanthin) [1]. Ascorbic acid, benzoic acids, flavonoids, flavonoid glycosides, dihydrochalcones, anthocyanins, proanthocyanidins and cinnamic acid derivatives are the main classes of antioxidant compounds found in apple fruits. Ascorbic acid content varies in wide ranges, e.g., 0.4–13.2 mg/100 g fresh fruit [12] or 13.1 mg/100 mL apple juice [19]. Gallic, *p*-hydroxybenzoic, protocatechuic and syringic acids were the main compounds from the benzoic acid class found in various apple cultivars [20,21,22,23]. Flavan-3-ol derivatives were represented by catechin and epicatechin, which were identified in apple fruits in many studies [20,21,22,23,24,25,26,27,28,29,30,31,32,33,34]. Other flavonoids were quercetin, myricetin, kaempferol, as well as epicatechin di-, tri-, tetramers and epigallocatechin [20,21,30]. Flavonoid glycosides were more important in all parts of apple fruits. Almost all were quercetin-based derivatives [1,20,21,22,23,24,25,26,28,29,31,32,33,34,35,36]. Dihydrochalcone derivatives were identified in apples even as aglycones such as phloretin [1,20,21,26,34] or especially as glycosides [1,22,24,25,29,30]. Other important antioxidant compounds found in apples belong to the cinnamic acid derivative class. Both hydroxycinnamic acids and cinnamic acid esters with (-)-quinic acid were identified [1,21,22,23,24,25,26,27,28,29,30,31,32,33,34,35]. Cyanidin-3-*O*-glucoside and cyanidin-3-*O*-galactoside from anthocyanidin class, procyanidin B1, B2, and their di- and trimers from procyanidin class were also identified in apples [1,21,22,23,24,25,26,27,29,30,32,33,34]. These antioxidant compounds have been identified even as individual compounds or as total polyphenols, flavonoids, along with the overall antioxidant activity of various fresh and unprocessed or processed apple cultivars.

Antioxidant activity of fruits and extracts is determined by various methods that include reactions of antioxidant compounds with specific reagents. In this regard, the DPPH· method (2,2-diphenyl-1-picrylhydrazyl) is widely used. The stable DPPH· radical has the ability to scavenge other radicals resulted by homolytic splitting of phenolic or enolic groups in antioxidant compounds [37,38]. The maximum absorbance of DPPH· at 517 nm is shifted to lower values for the neutral reaction products. The DPPH· method was also used in combination with Electron Paramagnetic Resonance (EPR) spectroscopy for direct detection of free radicals in various vegetables, spices and fruits, including apples [7]. Another method used for evaluation of antioxidant activity of fruits is the TEAC/ABTS·^+^ assay (Trolox equivalent antioxidant capacity/2,2′-azino-bis(3-ethylbenzthiazoline-6-sulfonic acid, radical cation)) [39,40]. FRAP (ferric reducing ability of plasma) of antioxidant compounds and extracts is determined by monitoring the absorbance at 593 nm [39]. The Folin–Ciocalteu assay allows measurement of the total reducing capacity, including the reaction with phenols and enols [39]. Alvarez and co-workers studied the overall antioxidant activity of apple juices through DPPH·, ABTS·^+^ and FRAP. The PCA analysis (principal component analysis) allowed differentiation of apple juices by geographical origin and type of juice [35]. The geographical origin was established for the “Golden Delicious” apple variety through DPPH·, ABTS·^+^ and FRAP methods by Fernández-Jalao and collaborators [24] or through TEAC and FRAP methods by Łysiak et al. for the “Jonagold” variety [29]. Similar studies were performed to evaluate the overall antioxidant activity of different apple cultivars by DPPH· and AEAC (ascorbate equivalent antioxidant capacity) [11], by FRAP technique for flesh and peel of different apple genotypes [25,28], by Folin–Ciocalteu, FRAP, DPPH· and ABTS·^+^ methods for flesh, peel and whole apple fruits of various cultivars [33,36,41,42], as well as through DPPH· and FRAP assays for the unripe “Fuji” apple variety at different growing periods [34]. Multivariate statistical analyses such as PCA and PLS (partial least squares/projection in latent structures) were generally used for classifying/grouping or discriminating of apple varieties, processed apples, or apple juices by means of antioxidant compound composition, total antioxidant activity, or spectroscopic methods (FTIR, MIR and NIR—Fourier transform-, middle- and near infrared spectroscopy, UV-Vis spectrophotometry or ^1^H-NMR—^1^H nuclear magnetic resonance) [35,43,44,45,46,47].

There are many research articles related to the site selection and characteristics, cultivars, soil and crop management, pesticide and pest management, harvest and post-harvest handling of organic apples, as well as consumer preferences [9,48,49,50,51]. However, aroma profile of apples from organic orchards, their authentication and processing levels were studied using PCA and PLS multivariate statistical analysis techniques [52,53,54,55,56]. Unfortunately, only a few studies were performed for discriminating between organic apples by means of their antioxidant properties. This is the case for the influence of freezing of organic and conventional apples on the polyphenol content and antioxidant activity. The discrimination of the samples by PLS-DA (discriminant analysis) was conducted [31]. On the other hand, the PCA discrimination of organic and conventional apples by means of the main nutrients (mineral contents and their ratios) [57] or organic apple juices as affected by processing, using antioxidant characteristics (ascorbic acid, polyphenols, total antioxidant capacity, particular antioxidant contents) and organoleptic properties [58] was also investigated. To our knowledge, no research on the application of DPPH· kinetics for the discrimination of organic/local apple varieties was performed.

The goal of the present study was to discriminate organic and non-organic (conventional) apple varieties cultivated in the western region of Romania using the DPPH· kinetics–PCA approach. This is a fast and simple approach based on the determination of the mean DPPH· reaction rates during specific time ranges and is for the first time applied for apple fruits.

## 2. Results and Discussion

### 2.1. Radical Scavenging Activity of Apple Extracts

The antioxidant activity of apple shell and core extracts of various varieties was evaluated as the ability to capture free radicals (radical scavenging activity, *RSA*, see Section 3.3). Methods of evaluation were based on the use of the stable free radical, 2,2-diphenyl-1-picrylhydrazyl (DPPH·), for which there is a significant hypsochromic displacement, from 517 nm for the free radical, to values lower than 450 nm (generally) for the DPPH-H and reaction products derived from the antioxidant compounds. As apple extracts show complex mixtures of compounds with antioxidant activity (mainly flavonoids and flavonoid glycosides—e.g., quercetin-3-*O*-rutinoside, phenolic acids—e.g., chlorogenic acid, procyanidins—e.g., (+)-catechin, dihydrochalcones—e.g., phloretin-2′-*O*-glucoside, or anthocyanidins and anthocyanins—e.g., cyanidin-3-*O*-glucoside), their reaction was monitored by recording the absorbance of DPPH· over time, taking into account that possible interferences of the mentioned antioxidant compounds are negligible because the molar absorbance and the concentration of these compounds in the extracts are much lower compared to those of the free radical. These variations in absorbance have an inverse logarithmic allure, more or less accentuated depending on the part of the fruit from which the extract was obtained (see Supplementary Materials, Figures S1–S4). Three specific time ranges were set according to the drift ratios for these pseudo-linear ranges in the *Absorbance* versus *Time* plots. The step-by-step procedure looks for the maximization of the drift “*1*”/drift “*2*” and drift “*2*”/drift “*3*” ratios. For apple varieties (shell and core samples) the drifts and their ratios vary within very large limits. In order to compare the samples, the same time ranges were selected, according to previous conditions: time range “*1*” of 0–30 s, time range “*2*” of 30–180 s, and time range “*3*” of 180–900 s. Representative actual *RSA* values were selected at approximately a quarter of the second and third time ranges (1 min and 5 min), as well as at the upper limits of these ranges (3 min and 15 min).

In general, “Golden Delicious” apple shell caused a more pronounced decrease in DPPH· absorbance in the first period of monitoring. Antioxidant activities were evaluated by *RSA* values at various monitoring times (Table 1 and Figure S1). Thus, for “Golden Delicious” shell extracts, the *RSA* values after 60 s were between 31.6 and 88.7%, while for the corresponding core extracts, they were generally lower (34.2–76.7%). The highest antioxidant activities were observed for the samples from organic orchard. For the shell extracts, the *RSA* values were 96.65 (±0.72)% and 95.25 (±0.27)%, at the end of monitoring (Table 1). The same trend is observed for the intermediate moments of time. In general, the antioxidant activity of “Golden Delicious” variety was 1–5% higher for the shell extracts, compared to the core ones, except for the samples from the supermarket (“*Gd*(*sh*)*_MK*” and “*Gd*(*co*)*_MK*”), where the differences were larger (Table 1).

To compare the antioxidant activity of apple extracts of various varieties, similar studies were performed for standards, natural or synthetic antioxidant compounds, such as resveratrol and propyl gallate. The variation of DPPH· absorbance in the presence of these antioxidant compounds of various concentrations was also inversely logarithmic, the most active being resveratrol at a concentration of 1 mM. It was observed that both resveratrol and propyl gallate show significant antioxidant activity even after 15 min of monitoring (Table S1). If the *RSA* values for “Golden Delicious” apple extracts are compared with those for the standard compounds mentioned above, it can be seen that the apple shell extracts are close in *RSA* value to the final *RSA* values for standard antioxidant compound resveratrol at a concentration of 1 mM, respectively, 0.2 mM propyl gallate (82.5–84.2%, Table S1).

For the “Florina” apple variety, the *RSA* variations were quite different for the shell samples, compared to the core ones, especially for those from the organic orchard (sample “*Fl*(*sh*)*_SRa*”) and from the non-organic orchard (harvested from the conventional orchard, “*Fl*(*sh*)*_LG*”) (Figure S2). The biggest differences between the antioxidant activities for the extracts from the shell, respectively, from the core, were observed especially at the beginning of the monitoring, at 1 and 3 min (Figure S2 and Table 2). For example, samples from Lugoj showed *RSA* values at 1 min of spectrophotometric monitoring of 89.09 (±1.95)% for apple shell extracts and only 45.86 (±2.17)% for core extracts, differences that remain important even at 3 min of monitoring: 92.98 (±0.40)% and, respectively, 70.2 (±1.56)% (Table 2). At the end of the analysis, the *RSA* values were quite close: 93.47 (±0.5)% and 90.85 (±4.92)% (higher than for the standard solution of 1 mM resveratrol, Table S1). The same behavior was observed for the organic samples from Şiria (especially samples “*Fl*(*sh*)*_SRa*” and “*Fl* (*co*)*_SRa*”), for which the *RSA* values at 1 min were 85.33 (±0.30)% for the shell and 64.43 (±2.52)% for the core, respectively, 93.45 (±0.06)% and 93.23 (±0.16)% at the end of the analysis (Table 2). Lower values were obtained both for the organic “*Fl*(*sh*)*_SRb*” and “*Fl*(*co*)*_SRb*” samples from Şiria, and especially for the samples from the supermarket (“*Fl*(*sh*)*_MK*” and “*Fl*(*co*)*_MK*”), for which the antioxidant activity in the core was very low (25.64 (±3.05)%, weaker even than the antioxidant activity of resveratrol at the lowest analyzed concentrations of 0.2 mM and 0.1 mM (for which the *RSA* values were 51.41–59.55%, Table S1).

The “Generos” apple variety showed interesting behavior in terms of antioxidant activity compared to the other varieties analyzed. The decrease in absorbance for “Generos” apple core extracts is significant even after 900 s of monitoring, and in the case of the organic “*Gn*(*co*)*_SR*” samples from Şiria it is even more accentuated for these apple core extracts. Quantification of this behavior by *RSA* (Figure S3 and Table 3) led to intermediate values at 3 min almost identical for the organic samples from Şiria (78.45 (±0.04)% and 78.13 (±3.08)% for the shell and core samples), and at the end of the analysis, 79.12 (±0.14)% and 94.69 (±0.27)% for the same extracts, values similar to those of 0.2 mM propyl gallate for the first case and higher than that of resveratrol at a concentration of 1 mM (see Table S1).

Behaviors similar to those for “Generos” apple shell and core extracts were also observed for the “Starkrimson” apple variety, all from organic orchard (samples “*SRa*” and “*SRb*”, Figure S4 and Table 4). Thus, if at 1 min of determination the *RSA* values for the shell samples from organic orchard (samples “*Sk*(*sh*)*_SRa*” and “*Sk*(*sh*)*_SRb*”) were 90.16 (±1.25)% and 91.78 (±0.1)%, respectively, for core samples 71.77 (±2.75)%, at the end of the monitoring they became very close: 93.00–94.71% for the shell samples, respectively, 96.63% for the core ones. These values are higher than the most active standard solutions of 1 mM resveratrol and 0.2 mM propyl gallate (Figure S4, Table 4 and Table S1). For the supermarket samples, the antioxidant activities for “Starkrimson” apple shell extracts were double compared to those for core extracts, throughout the spectrophotometric monitoring (48.37 (±1.57)% and 26.05 (±2.27)% at 1 min of monitoring, respectively, 90.39 (±0.65)% and 44.89 (±4.16)% at the end of the monitoring, Figure S4 and Table 4).

There are some studies regarding the overall antioxidant activity of apples and other fruits, including the DPPH· method. The antioxidant activity (measured by the DPPH· method and expressed as mg TE/L) of apple beverages from various geographical origins was well correlated with other antioxidant-related parameters such as total phenolic/flavonoid contents or the concentration of some specific antioxidant compounds [35]. Similar studies on the overall antioxidant activity allow clustering of apple cultivars by location or processing technology [20,24,59,60]. DPPH·-based methods were also used for evaluating the freely soluble and deeply bound antioxidants in organic apple juices [58]. These parameters, combined with organoleptic ones, allowed classification of organic apple juices according to pressing technology. Our previous studies revealed important antioxidant activities, according to DPPH· radical scavenging activity [61,62,63,64]. Pomegranate extracts were differentiated by DPPH·-based antioxidant activity according to the ethanol–water ratio or fruit parts [65,66]. On the other hand, various parts of kiwi and papaya fruits were differentiated through their DPPH· antioxidant activity [67,68]. Kiwi shell extracts had higher antioxidant activity than the core samples. Similarly, ripe papaya extracts were more active from this point of view.

### 2.2. DPPH· Kinetics Approach for the Apple Extracts

The behavior of apple extracts in the presence of free radicals can be easily evaluated to compare the various samples in terms of antioxidant activity, not only as the actual value (*RSA* values at various times). It can be achieved by studying the reaction kinetics of antioxidant compounds in extracts with free radical DPPH·. The variation of the concentration of DPPH· in time, in the presence of these antioxidant compounds, has pseudo-linear ranges, observed mainly on the time intervals 0–30 s (interval “*1*”), 30–180 s (interval “*2*”) and 180–900 s (interval “*3*”). These time ranges were selected according to drift ratios of successive ranges in the *Absorbance* versus *Time* plots, as was mentioned above (see Section 2.1). They were maximized with the restriction of havingthe same time range limits. Consequently, the following drift ratios were obtained, respectively: drift “*1*”/drift ”*2*” ratios of 5.8–18.8 and 2.5–16.3 for “Golden Delicious” shell and core samples, drift “*2*”/drift ”*3*” ratios of 3.0–101.4 and 5.9–40.7 for “Golden Delicious” shell and core samples; drift “*1*”/drift ”*2*” ratios of 18.7–127.9 and 3.9–37.5 for “Florina” shell and core samples, drift “*2*”/drift ”*3*” ratios of 5.4–138.3 and 4.8–41.2 for “Florina” shell and core samples; drift “*1*”/drift ”*2*” ratios of 11.6–33.0 and 6.5–9.9 for “Generos” shell and core samples, drift “*2*”/drift ”*3*” ratios of 26.0–52.9 and 6.0–7.5 for “Generos” shell and core samples; drift “*1*”/drift ”*2*” ratios of 5.4–170.7 and 7.5–9.6 for “Starkrimson” shell and core samples, drift “*2*”/drift ”*3*” ratios of 6.7–115.3 and 4.7–19.9 for “Starkrimson” shell and core samples.

The ratio of the variation of the DPPH· concentration (Δ*C_DPPH·_*, μM, variation with negative value due to the decrease in the concentration over time) to the considered time interval (Δ*t*, s) represents the mean reaction rate of DPPH· (${\bar{v}}_{1-3}$, μM/s, see Section 3.4) with antioxidant compounds in extracts. Antioxidant compounds belong to various structural classes and have various behaviors in reaction with free radicals. Thus, these mean DPPH· reaction rates were compared for the time intervals considered, for the extracts from various parts of the fruit, respectively, for standard solutions of natural and synthetic antioxidant compounds (resveratrol and propyl gallate).

**Figure 1:**
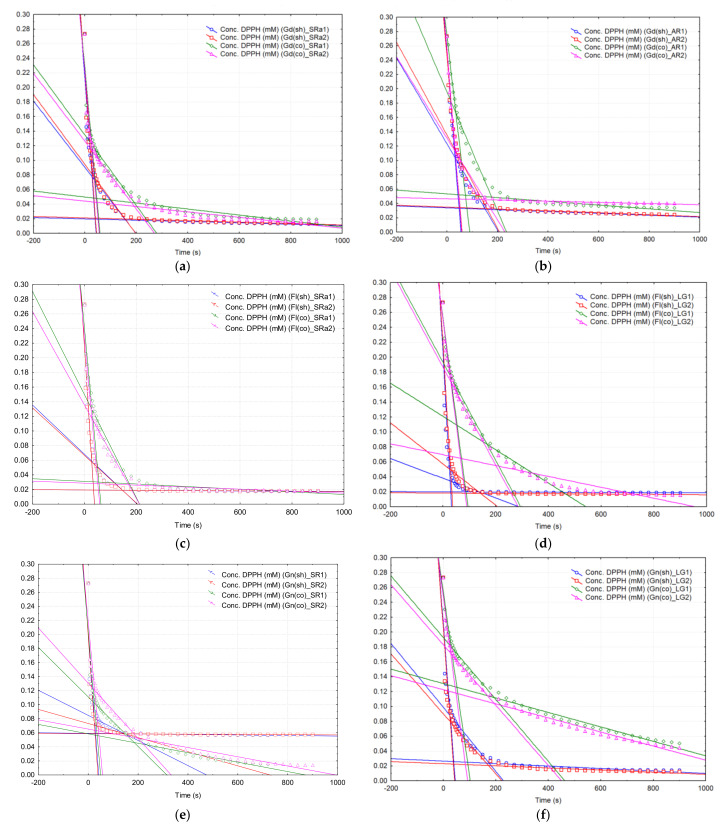

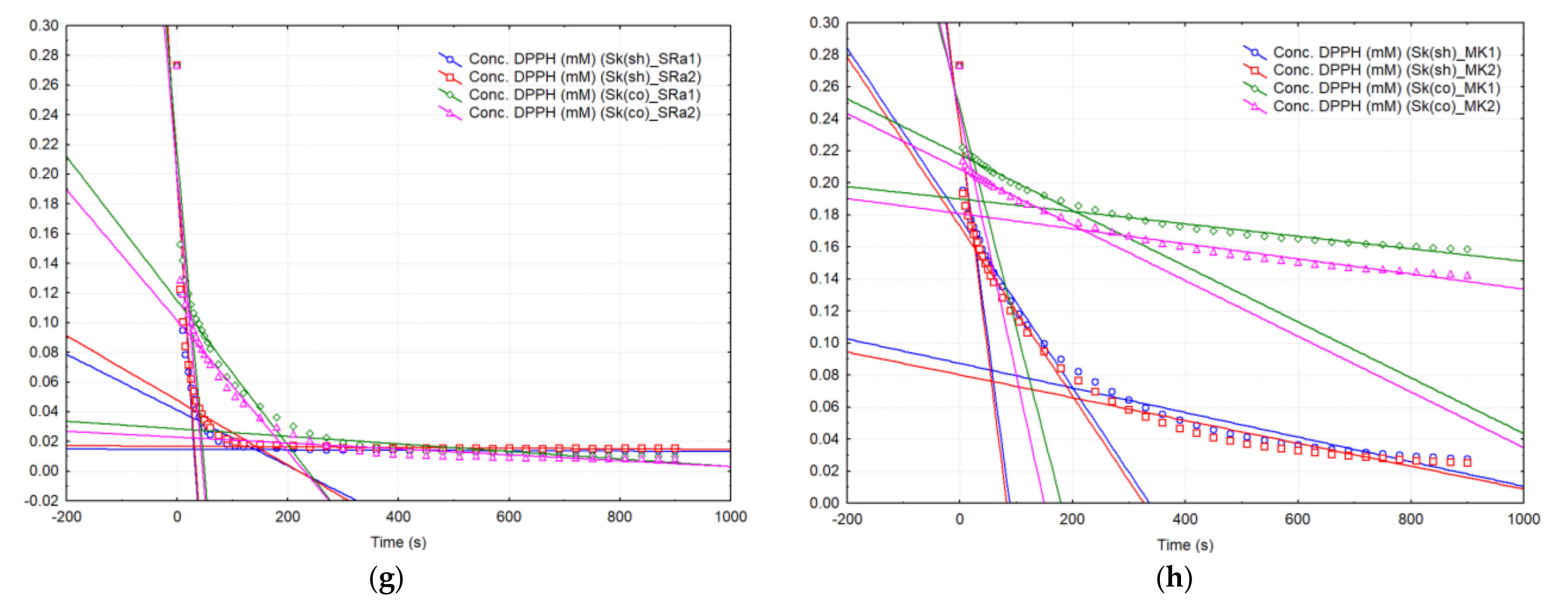
Variation of the DPPH· concentration during the reaction with antioxidant compounds from the extracts obtained from: (**a**) the organic shell and core of the “Golden Delicious” apple variety (*Gd*(*sh*)*_SRa* and *Gd*(*co*)*_SRa*); (**b**) the non-organic shell and core of the “Golden Delicious” apple variety (*Gd*(*sh*)*_AR* and *Gd*(*co*)*_AR*); (**c**) the organic shell and core of the “Florina” apple variety (*Fl*(*sh*)*_SRa* and *Fl*(*co*)*_SRa*); (**d**) the non-organic shell and core of the “Florina” apple variety (*Fl*(*sh*)*_LG* and *Fl*(*co*)*_LG*); (**e**) the organic shell and core of the “Generos” apple variety (*Gn*(*sh*)*_SR* and *Gn*(*co*)*_SR*); (**f**) the organic shell and core of the “Generos” apple variety (*Gn*(*sh*)*_LG* and *Gn*(*co*)*_LG*); (**g**) the organic shell and core of the “Starkrimson” apple variety (*Sk*(*sh*)*_SRa* and *Sk*(*co*)*_SRa*); (**h**) the non-organic shell and core of the “Starkrimson” apple variety (*Sk*(*sh*)*_MK* and *Sk*(*co*)*_MK*) (all determinations are presented as duplicates “*1*” and “*2*”). See Supplementary Materials for all DPPH· kinetic results.

The mean DPPH· reaction rates in the presence of the “Golden Delicious” apple shell extracts were generally higher for the first time interval, compared to the case of core extracts, the values being between 4.25 and 5.65 μM/s and 2.1 and 4.8 μM/s, respectively, for the organic and non-organic samples harvested directly from the orchards (samples “*Gd*(*sh*)*_SRa*”/“*Gd*(*co*)*_SRa*”, “*Gd*(*sh*)*_SRb*”/“*Gd*(*co*)*_SRa*”, “*Gd*(*sh*)*_AR*”/“*Gd*(*co*)*_AR*” and “*Gd*(*sh*)*_LG*”/“*Gd*(*co*)*_LG*”, Table 5, Figure 1a,b and Figure S5), while for the samples from the supermarket, the values of these rates were quite close, 1.75 (±0.07) μM/s and 2.05 (±0.64) μM/s (samples “*Gd*(*sh*)*_MK*” and “*Gd*(*co*)*_MK*”, Table 5, Figure S5). These values were reversed or at most very close for the second interval, where the DPPH· rates were in the range 0.13–0.85 μM/s (Table 5). With the exception of samples from the supermarket, the mean DPPH· reaction rates over the third time interval were higher for the core samples, compared to those in the shell (0.017–0.051 μM/s and 0.003–0.013 μM/s, respectively), indicating a prolonged antioxidant effect in the first case (even if the absolute *RSA* value was higher for the shell samples). These values were consistent with those obtained for standard antioxidant solutions, for which the mean DPPH· reaction rates on the first interval were between 2.6 and 4.2 μM/s, for the second interval between 0.06 and 0.30 μM/s and only 0.014 and 0.057 μM/s for the third interval (Table S18).

For “Florina” shell and core extracts, the mean DPPH· reaction rates showed similar comparative values to those for the first time interval. These were higher or equal for the extracts from the two parts of the apple, being between 3.75 and 6.10 μM/s for the extracts from the shell and 0.95 and 3.75 μM/s for those from the core (Figure 1c,d and Figure S6, Table 6). For the second and third time intervals, the mean DPPH· reaction rates were generally higher for the core extracts, compared to those in the shell (0.03–0.30 μM/s for the shell and 0.09–0.70 μM/s for core, in the case of the second interval, respectively, 0.001–0.028 μM/s and 0.016–0.136 μM/s, Table 6), highlighting the antioxidant activity even after these monitoring periods.

For the “Generos” variety, the largest differences resulting from the kinetic analysis were observed, especially in the case of the mean DPPH· reaction rates for the third time interval (Table 7). The values of these rates for the “Generos” apple samples from the non-organic orchard from Lugoj were only 0.015 (±0.002) μM/s for the shell extracts and more than three times higher for the core ones (0.053 (±0.062) μM/s, Table 7), while for the organic samples from Şiria (samples “*Gn*(*sh*)*_SR*” and “*Gn*(*co*)*_SR*”) these differences were even greater: 0.003 (±0.002) μM/s for the shell extracts and 0.066 (±0.001) μM/s for those of the “Generos” apple core (Table 7).

When comparing the DPPH· kinetics of the “Starkrimson” apple extracts for the organic samples from Şiria, respectively, from the supermarket, a similar behavior was found to the organic ones. Thus, the organic samples from Şiria showed significant rates on the first time interval for “Starkrimson” apple shell extracts (5.9 μM/s, Figure 1g, Figure S6 and Table 8), and for the core extracts of 4.3 μM/s (Figure 1g and Table 8). On the other hand, these values were lower for the samples in the supermarket (2.7 (±0.14) μM/s and 1.5 (±0.14) μM/s, respectively, Figure 1h, Figure S6 and Table 8). For the third interval, only in the case of organic “Starkrimson” apple extracts were higher values found for the reaction rates corresponding to the core extracts, compared to those of the shell (0.001–0.002 μM/s for shell extracts and 0.023 (±0.004) μM/s for core extracts from “Starkrimson” apples from Şiria, Table 8). Antioxidant activity becomes more important in this latest time range for both types of “Starkrimson” apple extracts in the supermarket (0.043–0.074 M/s, Table 8).

The previous DPPH· kinetic approach was only applied for specific antioxidant compounds such as synthetic *tert*butylhydroxytoluene [69], ascorbic acid, tocopherols, caffeic, ferulic, chlorogenic, sinapic, vanillic, genistic and gallic acids, gallate esters, quercetin, epicatechin, sesamol and curcumin [37,63,70,71,72,73] or lemon and pomegranate juices, green tea infusion, purple corn and red-fleshed sweet potato, tomato, pomegranate and kiwi extracts, rosemary essential oil [66,68,74,75,76]. Up to now, no literature data exist on the DPPH· kinetic and PCA classification on organic apple samples. Only a DPPH·-based kinetic model of apple juice enzymatic browning in the presence of cyclodextrins has been investigated [77]. In the present study, a qualitatively correlation between the DPPH· kinetic results and biologically active compound classes contained in various parts of apple samples or as a function of the orchard locations can be performed. In all cases the apple shell samples reveal higher DPPH· reaction rates on the first time range. This means that apple shall samples have a higher content of antioxidant compounds with higher reactivity such as ascorbic acid or flavonoids, dihydrochalcones and cinnamic acid derivatives with less hindered phenolic hydroxyl groups. This observation can also be made for apples obtained from organic orchards, even the locations with approximately the same altitudes (109 m for the organic orchard and 117–124 m for non-organic orchards).

### 2.3. Correlations and Principal Component Analysis (PCA) of the Antioxidant Activity and DPPH· Kinetics of Apple Extracts

The overall antioxidant activity, quantified by *RSA* values at various times or the DPPH· kinetics parameters, were subjected to linear regression analysis and multivariate statistical analysis. Both statistic procedures allow identification of the significant parameters for differentiation between samples. Thus, linear correlations between the *RSA* values and mean DPPH· reaction rates provide very good results, especially for the mean DPPH· reaction rate for the first time range, ${\bar{v}}_{1}$. The correlation with the *RSA* at 1 min had the best statistic parameters. The correlation coefficient of *r*^2^ = 0.982 was the same even for all datasets (*n* = 54 organic and non-organic apple samples) or for organic apple samples (*n* = 26) (see Equations (1) and (2), as well as graph plots and linear equations in Figures S7 and S9). Correlations with the *RSA* at other times were also statistically significant, even the correlation coefficients were slightly lower (*r*^2^ of 0.901 for *RSA* at 3 min, 0.825 for *RSA* at 5 min and 0.718 for *RSA* at 15 min, *n* = 54, Figure S7). On the other hand, mean DPPH· reaction rates on the second and third time ranges do not correlate with *RSA*, neither for all dataset, nor for the dataset corresponding to the organic apples (Figures S8 and S9).

(1) $${\bar{v}}_{1}=-0.38(\pm 0.08)\;+\;0.068(\pm 0.001)\;\cdot \;RSA(1\prime )$$

*n* = 54, *r*^2^ = 0.982, *F* = 2855, *s* = 0.20, *p* < 0.00001.

(2) $${\bar{v}}_{1}=-0.90(\pm 0.22)\;+\;0.0074(\pm 0.003)\;\cdot \;RSA(1\prime )$$

*n* = 26, *r*^2^ = 0.982, *F* = 632, *s* = 0.16, *p* < 0.00001.

The classical analyses of these apple varieties (*n* = 26 core samples and *n* = 12 organic core samples) were performed in an earlier study and consist of *Moisture* (g/100 g fresh weight), *Minerals* (g/100 g fresh weight), *pH*, *Sugar content* (° Brix), *Total polyphenols* (ppm), as well as specific element contents (such as Cu, Cr, Zn, Fe, Mn, K, or P, in ppm). The attempt to correlate antioxidant and kinetic parameters with the above-mentioned ones provides interesting results for the *RSA*(*3′–15′*) versus *pH* (the correlation coefficient, *r*, was 0.41 for the *RSA* (*15′*) parameter; for all apple core samples, *n* = 26). On the other hand, the mean DPPH· reaction rate on the second time range, ${\bar{v}}_{2}$, correlates well with the *pH* parameter (*r* = 0.52, *n* = 26). Valuable correlations were obtained if the organic samples were considered. Thus, *Sugar content* (° Brix) correlates well with almost all *RSA* and DPPH· kinetic parameters (*RSA*(*1′–15′*) versus *Sugar content*, *r* = 0.64–0.88, *n* = 12; ${\bar{v}}_{1}$ versus *Sugar content*, *r* = 0.92, *n* = 12). These observations suggest that the high content of antioxidants with glycoside groups (one of the most important antioxidant class in apples—e.g., flavonoid glycosides, dihydrochalcone glycosides as well as anthocyanins) that can hydrolyze to free sugars agreed with the high *RSA* values. Moreover, high acidic condition (lower *pH*) reduces the overall antioxidant activity (see Supplementary Materials for linear correlations between *RSA* or DPPH· kinetic parameters and *pH* or *Sugar content*, Equations (S1)–(S9)).

Due to partial intercorrelations between *RSA* and mean DPPH· reaction rates (i.e., ${\bar{v}}_{1}$), the multivariate statistical analysis techniques can process the big dataset by removing the inconvenience of overlapping. Principal component analysis (PCA) uses the maximum variance of the data in order to transform the original axes (parameters) into new axes named principal components (*PCs*) or factors. The *PCs* are orthogonal and the intercorrelations are removed. The graph representation of the translation coordinates provides the scores plot and reveals the similarity/dissimilarity between cases (the grouping of the samples). On the other hand, the graph representation of the cosines of the rotational angles provides the loadings plot, which reveals the importance of the original parameters to the classification.

In the case of organic and non-organic apples, shell and core extracts were considered as cases, while the original parameters were *RSA* and mean DPPH· reaction rates at considered times. The attempt to use apple variety as discrimination parameter does not provide clear classifications of the samples, as is presented in Figure S10 for *PC*_2_ versus *PC*_1_ scores plot. However, almost all “Golden Delicious” and “Starkrimson” varieties are grouped in the central-left region of the *PC*_2_ versus *PC*_1_ scores plot (similar grouping can be observed in the *PC*_3_ versus *PC*_1_ scores plot in Figure S11). All *RSA* parameters are significant for the *PC*_1_, as well as the mean DPPH· reaction rate on the first time range, ${\bar{v}}_{1}$. On the other hand, the mean DPPH· reaction rates ${\bar{v}}_{2}$ and ${\bar{v}}_{2}$ are significant for the *PC*_2_ in the PCA analysis. The first two *PCs* explain 92.16% of the variance of the antioxidant activity and DPPH· kinetics data (67.60% for *PC*_1_ and 24.56% for *PC*_2_, Figure S11).

**Figure 2:**
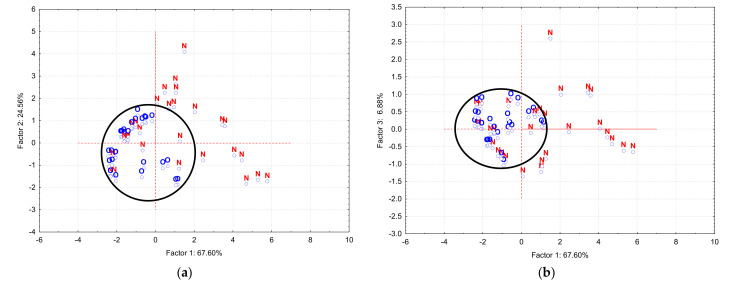
PCA results for the antioxidant activity and DPPH· kinetics data of all organic (“*O*”—blue) and non-organic (“*N*”—red) apple extracts: (**a**) *PC*_2_ versus *PC*_1_ scores plot; (**b**) *PC*_3_ versus *PC*_1_ scores plot.

The PCA approach was then focused to the discrimination between apple parts (coded as “*S*” for shell and “*C*” for core of the apple in PCA). The shell cases were more grouped in the center of the *PC*_2_ versus *PC*_1_ and *PC*_3_ versus *PC*_1_ scores plots, while core samples had a larger distribution (Figure S12). Good results were obtained if the organic (“*O*”) and non-organic (“*N*”) samples were considered in PCA. In both *PC*_2_ versus *PC*_1_ and *PC*_3_ versus *PC*_1_ scores plots, organic apple samples are well grouped in the center of these plots. They are more similar than non-organic samples, which have a wide distribution (Figure 2). The influence of *RSA* and ${\bar{v}}_{1}$ for *PC*_1_, as well as ${\bar{v}}_{2}$ and ${\bar{v}}_{3}$ for *PC*_2_ are the same such as for the above-mentioned classifications.

Taking into account these classifications based on the type of orchard, PCA was applied for every apple variety. Organic “Golden Delicious” apple cases were clearly grouped in the center-right of the both *PC*_2_ versus *PC*_1_ and *PC*_3_ versus *PC*_1_ scores plots (Figure S13a,b). Only few non-organic cases are located in this region. The classification along *PC*_1_ is mainly based on all *RSA* values (with a slight influence for PC_2_ in the case of *RSA* (*1′*) and *RSA* (*15′*)), as well as mean DPPH· reaction rates on the first and third time ranges, ${\bar{v}}_{1}$ and ${\bar{v}}_{3}$. Almost 93.4% of the variance of the data are explained by *PC*_1_ and *PC*_2_ (Figure S14a,b). Similar results were obtained for the “Florina” variety, where organic samples are located in the center of the scores plots (94.11% explained variance for the *PC*_1_ and *PC*_2_, Figure S13c,d for scores plots, Figure S14c,d for loadings plots and eigenvalues of the correlation matrix), as well as for the “Generos” and “Starkrimson” apple varieties, with locations in the center or center-right of the scores plots (Figure S13e–h explains variances of 92.53% and 97.70% for the first two *PCs*, respectively; Figure S14e–h).

PCA approach allows partial differentiation of the organic and non-organic apples, especially through the DPPH· kinetics parameters. Kinetics for the first time interval of the DPPH· reaction especially influence the *PC*_1_ (together with *RSA* parameters), while the kinetic parameters for the next time ranges are important for *PC*_2_. This discrimination can be due to the different antioxidant composition of the apple, such as the antioxidant compounds with higher reactivity that are more concentrated in organic samples (e.g., ascorbic acid, phenolic compounds with less hindered hydroxyl groups). However, DPPH·–apple antioxidant compound interaction involves very complex parallel and consecutive reactions and it is difficult to evaluate such aspects if the study is focused on the specific compounds. These affirmations are sustained by the research on the ascorbic acid, total phenolic and flavonoid, as well as specific antioxidant contents in apples and apple juices of various geographical origin [35]. The ascorbic acid content in such samples ranged from 140 mg/L in Europe to 247 mg/L in North America, and from 148 mg/L in clarified juice to 254 mg/L for integral apple juice. Chlorogenic acid is the most concentrated in the last type of apple beverage, but all identified antioxidant compound contents vary in wide ranges. These samples were relatively well grouped by PCA, with a significant influence of the chlorogenic acid content. Similar research related to the geographical origin or location of apples was performed for “Golden Delicious” and “Jonagold” varieties, where the same chlorogenic acid was the most concentrated [24,29]. PCA was applied for organic apple classification or authentication especially through the aroma compound profile or other organoleptic and image processing parameters [55,56]. Regarding the antioxidant activity of organic apples, PCA or PLS were only applied for differentiation of apples subjected to freezing pre-treatments or processing [31,58].

## 3. Materials and Methods

### 3.1. Fruits and Chemicals

Four apple varieties cultivated and/or commercialized in the western region of Romania (year 2019) were considered for this study. Thus, 5 samples of the “Golden Delicious” variety (code “*Gd*”) were obtained from the organic orchard (2 samples harvested from opposite locations of the orchard) and conventional orchards (3 samples from which 2 samples were obtained directly from the orchards and 1 sample from the local supermarket). “Florina” apples (code “*Fl*”) were obtained from the same organic orchard (2 samples) and from conventional orchards (1 sample directly from the orchard and 1 sample from the local supermarket). Two samples of the “Generos” variety (code “*Gn*”) were obtained from the above-mentioned organic orchard (1 sample) and directly from the conventional orchard (1 sample). Finally, the “Starkrimson” variety (code “*Sk*”) was obtained from the organic orchard (2 samples) and from the local supermarket (1 sample). Codes, description and other information related to the apple samples used in this study are presented in Table 9. The organic orchard is located in western Romania (Şiria, Arad county, 46°16′2″ N, 21°38′18″ E, altitude 109 m). Only manure was used as fertilizer and no pesticides were applied. Apples were harvested at the maturity stage and they were of medium size (specific for every variety). Four fruits were used for every sample. Fresh apples were stored at 4 °C until the solid–liquid extraction, which was performed up to two days after collection.

To determine the antioxidant activity, evaluated by the free radical scavenging activity (*RSA*), the stable free radical 2,2-diphenyl-1-picrylhydrazyl (DPPH·, >99%, Merck & Co., Inc., Kenilworth, NJ, USA) was used. Ethanol (volumetric concentration of 96%, *pro analysis* grade, Chimopar, Bucharest) was used for the extraction and *RSA* evaluation. Polyphenols used as standard antioxidant compounds were of high purity as follows: resveratrol 98% (J & K Scientific LLC, San Jose, CA, USA) and propyl gallate ≥98% (Sigma-Aldrich Co., St. Louis, MO, USA).

### 3.2. Obtaining of Apple Extracts

Both shell and core (abbreviated as “*sh*” and “*co*”, Table 9) of the fruit were subjected to the extraction and analysis. They were manually separated using a knife. The apple shell had a thickness of 1–2 mm. Fresh samples were immediately milled using a 400 mL ceramic mortar and weighted for extraction. The solid–liquid extraction was performed under mild conditions (room temperature, in the dark) in order to reduce the thermal and oxidative degradation of antioxidant compounds. Thus, 5.0 g of ground sample was mixed with 20 mL of 96% ethanol in a 250 mL hermetically sealed extraction flask maintained at 25 (±1) °C in the dark. The mixture was intermittently stirred for 24 h. Then, the extract was filtered at normal pressure using filter paper of 5–8 μm pore size and 87 g/m^2^ basis weight, washed with 2 mL of ethanol and centrifuged at 3000 rpm for 10 min at room temperature using a Heraeus AG centrifuge (Hanau, Germany). After decantation, the apple extract was diluted to 25 mL with ethanol in order to compare the results. They were stored at 4 °C until analysis. All extracts were obtained as duplicates (coded as “*1*” and “*2*”).

### 3.3. Evaluation of the Radical Scavenging Activity (RSA) by DPPH· Method

The overall antioxidant activity was monitored for 15 min by means of the *RSA* values, both for apple extracts and standard antioxidant compound solutions (resveratrol as natural antioxidant and propyl gallate as synthetic antioxidant). Thus, 0.5 mL of apple extracts or standard antioxidant solutions at various concentrations were mixed with 2 mL ethanol and a 0.5 mL 0.1 mM DPPH· ethanolic solution in a 10 mm quartz cuvette (the apple extract:DPPH· solution:ethanol volume ratio was 1:1:4). The mixture was immediately sealed and subjected to spectrophotometrical monitoring at 517 nm for 15 min. A CamSpec M501 UV-Vis spectrophotometer (CamSpec Ltd., Cambridge, UK) was used. Acquisition and handling of the UV-Vis data were performed by the *Time Scan Measurement* module for evaluation of *RSA* and DPPH· kinetics, and *Wavelength Scan Measurement* module for obtaining the DPPH· calibration curve, both from UV-Vis Analyst version 4.67 software (CamSpec Ltd., Cambridge, UK). The actual *RSA* values were obtained according to Equation (3), where *A*_*t* = 0_ and *A_t_* stand for absorbance at the start of the measurement (*t* = 0 s) and the time *t*.

(3) $$RSA=\frac{A_{t=0}-A_{t}}{A_{t=0}}\cdot 100$$

### 3.4. Evaluation of the DPPH· Kinetics

Due to the complex mixture of the apple extracts, the DPPH· interaction involves many antioxidant compounds in parallel, consecutive and competitive chemical reactions. They especially are phenolic and enolic compounds (e.g., flavonoid glycosides and ascorbic acid). As a consequence, it is difficult to evaluate the DPPH· kinetics in relation with a specific compound. This was the reason to use the mean DPPH· reaction rates for characteristic time ranges from the *Concentration* versus *Time* plots. Only DPPH· has significant absorbance at 517 nm in the apple extract–DPPH· solution mixture. During the reaction, DPPH· is converted into the neutral DPPH-H compound (2,2-diphenyl-1-pycrylhydrazine), which as significant absorbance at wavelengths lower than 450 nm. Neither antioxidant compounds in apple extracts, nor the reaction products have absorbance at 517 nm. Thus, the variation of the absorbance at 517 nm can be converted as the variance of DPPH· concentration in time. The *Concentration* versus *Absorbance* calibration curve for DPPH· solutions in the range of 0–300 μM was obtained with statistically significant parameters (*n* = 5, *r*^2^ = 0.999 and *p* < 0.000001). The DPPH· concentration in Equation (4) is expressed as μM and the absorbance *A* is determined at 517 nm. Generally, the *Concentration* versus *Time* plots reveal three pseudo-linear ranges. In order to compare the DPPH· kinetics results, these time ranges were set as 0–30 s for Δ*t*_1_, 30–180 s for Δ*t*_2_ and 180–900 s for Δ*t*_3_. The variation (decrease) in the DPPH· concentration for these three time ranges allows determination of the mean DPPH· reaction rate (Equation (5)) as the drift of the linear equation resulted from the *Concentration* versus *Time* correlation for every time range. In order to easier compare the DPPH· reaction rate values, they were expressed in μM/s.

(4) $$C_{DPPH}=159.58\left(\pm 2.80\right)\cdot A$$

(5) $${\bar{v}}_{1-3}=-\frac{\Delta C_{DPPH\left(1-3\right)}}{\Delta t_{1-3}}$$

### 3.5. Statistical, Correlational and Principal Component Analyses

All results were presented as means (±standard deviation, SD), obtained using the *Basic Statistics & Tables* and *One-way ANOVA* modules in Statistica 7.1 software (StatSoft, Inc., Tulsa, OK, USA). The same *One-way ANOVA* module was used for evaluating the significant differences between values by means of Tukey’s HSD (honest significant difference) test. Linear correlations for the DPPH· calibration curve, for evaluation of mean DPPH· reaction rates, as well as for the correlation between antioxidant activity and DPPH· kinetics parameters, were performed using *Multiple Linear Regression* module in the same Statistica package. Linear regression equations were statistically evaluated by means of the Pearson correlation coefficient, *r*, or determination coefficient, *r*^2^, *F*-Fisher and *p*-values, standard error of estimate, *s*, and standard errors for linear regression coefficients. The confidence limit and significance level were 0.95 and 0.05, respectively. Discrimination between sample types was based on the overall antioxidant activity and DPPH· kinetics parameter values. The data matrix was subjected to principal component analysis (PCA), which is a powerful multivariate statistical analysis technique that allows extraction of the useful information from a large dataset. In the DPPH· kinetics–PCA approach all samples were considered as cases while *RCA* and mean DPPH· reaction rates were set as variables. The scores and loadings plots provide information about the grouping of cases and the influence of variables to the classification, respectively. PCA was performed using a cross-validation method and centered data by means of the *Principal Components & Classification Analysis* module in the same Statistica 7.1 package.

## 4. Conclusions

The DPPH· kinetics—PCA approach was for the first time performed for discrimination between organic and non-organic fruits. This approach was successfully applied for organic and non-organic “Golden Delicious”, “Florina”, “Generos” and “Starkrimson” apple varieties cultivated in the western region of Romania. All apple samples reveal high antioxidant activity, according to the DPPH· method. However, DPPH· kinetics provide more important information that can be correlated with the type of orchard and the part of the fruit. The mean DPPH· reaction rates at the start of the reaction with the complex mixture of antioxidant compounds from apples are higher in organic samples, more evident for the outer part of the fruit. On the contrary, the apple cores have significant activity even at the later time, providing a prolonged antioxidant effect. As a conclusion, there are different antioxidant compound profiles in organic and non-organic apples, as well as according to the fruit part, which can be used for evaluating the quality of fruits through a relatively simple and fast combined technique.

## Figures and Tables

**Table 1:** Radical scavenging activity (*RSA*%) at various times of monitoring for the extracts obtained from organic and conventional “Golden Delicious” apple varieties. Values are expressed as means (±standard deviation, SD). In a column, values with different superscript letters are significantly different, according to the Tukey HSD (honest significant difference) test (*p* < 0.05). All *p*-level values are presented in the Supplementary Materials (Tables S2–S5).

Code	Organic or Non-Organic Orchard ^1^	*RSA* (1 min) (%)	*RSA* (3 min) (%)	*RSA* (5 min) (%)	*RSA* (15 min) (%)
*Gd*(*sh*)*_SRa*	*O*	78.76 (±0.67) ^a^	92.13 (±0.29) ^a^	93.68 (±0.35) ^a^	95.25 (±0.27) ^a^
*Gd*(*co*)*_SRa*	*O*	63.95 (±2.21) ^a^	80.41 (±1.34) ^a^	87.56 (±1.29) ^a^	93.43 (±0.62) ^a^
*Gd*(*sh*)*_SRb*	*O*	88.65 (±1.45) ^a^	95.66 (±0.80) ^a^	96.24 (±0.76) ^a^	96.65 (±0.72) ^a^
*Gd*(*co*)*_SRb*	*O*	76.68 (±1.54) ^a^	91.36 (±0.64) ^a^	94.31 (±0.47) ^a^	95.20 (±0.28) ^a^
*Gd*(*sh*)*_AR*	*N*	69.26 (±2.62) ^a^	87.13 (±0.65) ^a^	89.02 (±0.17) ^a^	91.13 (±0.05) ^a^
*Gd*(*co*)*_AR*	*N*	58.36 (±13.23) ^a^	79.97 (±3.97) ^a^	84.17 (±0.18) ^a^	86.51 (±1.70) ^a^
*Gd*(*sh*)*_LG*	*N*	82.83 (±8.27) ^a^	89.35 (±2.14) ^a^	90.19 (±1.75) ^a^	90.78 (±1.01) ^a^
*Gd*(*co*)*_LG*	*N*	36.72 (±0.54) ^b^	67.55 (±0.23) ^b^	79.77 (±0.22) ^a^	86.90 (±0.84) ^a^
*Gd*(*sh*)*_MK*	*N*	31.64 (±0.43) ^b^	42.29 (±0.99) ^c^	48.80 (±1.27) ^b^	60.37 (±1.21) ^b^
*Gd*(*co*)*_MK*	*N*	34.21 (±13.6) ^b^	39.13 (±15.72) ^c^	41.59 (±17.03) ^b^	45.52 (±18.64) ^b^

^1^ *O*—organic orchard; *N*—non-organic orchard; ^a,b,c^ Values with different superscript letters in a column are significantly different, according to the Tukey HSD test (*p* < 0.05).

**Table 2:** Radical scavenging activity (*RSA*%) at various times of monitoring for the extracts obtained from organic and conventional “Florina” apple varieties. Values are expressed as means (±standard deviation, SD). In a column, values with different superscript letters are significantly different, according to the Tukey HSD (honest significant difference) test (*p* < 0.05). All *p*-level values are presented in the Supplementary Materials (Tables S6–S9).

Code	Organic or Non-Organic Orchard ^1^	*RSA* (1 min) (%)	*RSA* (3 min) (%)	*RSA* (5 min) (%)	*RSA* (15 min) (%)
*Fl*(*sh*)*_SRa*	*O*	85.33 (±0.30) ^a^	92.47 (±0.02) ^a^	93.28 (±0.08) ^a^	93.45 (±0.06) ^a^
*Fl*(*co*)*_SRa*	*O*	64.43 (±2.52) ^b^	86.49 (±0.65) ^a^	91.28 (±0.14) ^a^	93.23 (±0.16) ^a^
*Fl*(*sh*)*_SRb*	*O*	61.25 (±0.49) ^b^	62.26 (±0.70) ^a,b^	62.29 (±0.67) ^b^	62.43 (±0.68) ^a^
*Fl*(*co*)*_SRb*	*O*	63.70 (±1.64) ^b^	68.90 (±1.70) ^a,b^	71.59 (±1.76) ^a,b^	74.98 (±0.72) ^a^
*Fl*(*sh*)*_LG*	*N*	89.09 (±1.95) ^a^	92.98 (±0.40) ^a^	93.33 (±0.48) ^a^	93.47 (±0.50) ^a^
*Fl*(*co*)*_LG*	*N*	45.86 (±2.17) ^b^	70.20 (±1.56) ^a,b^	81.89 (±1.15) ^a^	90.85 (±4.92) ^a^
*Fl*(*sh*)*_MK*	*N*	64.41 (±10.66) ^b^	71.16 (±13.00) ^a,b^	74.14 (±14.02) ^a^	79.30 (±15.62) ^a^
*Fl*(*co*)*_MK*	*N*	16.64 (±3.04) ^c^	20.21 (±3.49) ^c^	22.41 (±3.05) ^c^	25.64 (±3.05) ^b^

^1^ *O*—organic orchard; *N*—non-organic orchard; ^a,b,c^ Values with different superscript letters in a column are significantly different, according to the Tukey HSD test (*p* < 0.05).

**Table 3:** Radical scavenging activity (*RSA*%) at various times of monitoring for the extracts obtained from organic and conventional “Generos” apple varieties. Values are expressed as means (±standard deviation, SD). In a column, values with different superscript letters are significantly different, according to the Tukey HSD (honest significant difference) test (*p* < 0.05). All *p*-level values are presented in the Supplementary Materials (Tables S10–S13).

Code	Organic or Non-Organic Orchard ^1^	*RSA* (1 min) (%)	*RSA* (3 min) (%)	*RSA* (5 min) (%)	*RSA* (15 min) (%)
*Gn*(*sh*)*_SR*	*O*	75.32 (±1.37) ^a^	78.45 (±0.04) ^a^	78.69 (±0.06) ^a^	79.12 (±0.14) ^a^
*Gn*(*co*)*_SR*	*O*	64.81 (±4.56) ^a^	78.13 (±3.08) ^a^	84.89 (±2.21) ^b^	94.69 (±0.27) ^b^
*Gn*(*sh*)*_LG*	*N*	75.80 (±2.09) ^a^	89.56 (±1.05) ^a^	93.00 (±0.74) ^c^	95.19 (±0.49) ^b^
*Gn*(*co*)*_LG*	*N*	41.28 (±2.03) ^b^	55.88 (±2.37) ^b^	64.33 (±1.89) ^d^	82.78 (±1.53) ^c^

^1^ *O*—organic orchard; *N*—non-organic orchard; ^a,b,c,d^ Values with different superscript letters in a column are significantly different, according to the Tukey HSD test (*p* < 0.05).

**Table 4:** Radical scavenging activity (*RSA*%) at various times of monitoring for the extracts obtained from organic and conventional “Starkrimson” apple varieties. Values are expressed as means (±standard deviation, SD). In a column, values with different superscript letters are significantly different, according to the Tukey HSD (honest significant difference) test (*p* < 0.05). All *p*-level values are presented in the Supplementary Materials (Tables S14–S17).

Code	Organic or Non-Organic Orchard ^1^	*RSA* (1 min) (%)	*RSA* (3 min) (%)	*RSA* (5 min) (%)	*RSA* (15 min) (%)
*Sk*(*sh*)*_SRa*	*O*	90.16 (±1.25) ^a^	94.04 (±0.57) ^a^	94.52 (±0.48) ^a^	94.71 (±0.39) ^a^
*Sk*(*co*)*_SRa*	*O*	71.77 (±2.75) ^b^	87.97 (±1.73) ^a^	93.67 (±1.03) ^a^	96.63 (±0.42) ^a^
*Sk*(*sh*)*_SRb*	*O*	91.78 (±0.10) ^a^	92.71 (±0.44) ^a^	92.89 (±0.95) ^a^	93.00 (±1.37) ^a^
*Sk*(*sh*)*_MK*	*N*	48.37 (±1.57) ^c^	68.23 (±1.47) ^b^	77.59 (±1.52) ^b^	90.39 (±0.65) ^a^
*Sk*(*co*)*_MK*	*N*	26.05 (±2.27) ^d^	32.71 (±2.56) ^c^	36.77 (±3.07) ^c^	44.89 (±4.16) ^b^

^1^ *O*—organic orchard; *N*—non-organic orchard; ^a,b,c,d^ Values with different superscript letters in a column are significantly different, according to the Tukey HSD test (*p* < 0.05).

**Table 5:** Values of the mean DPPH· reaction rates (${\bar{v}}_{1},\;{\bar{v}}_{2}$ and ${\bar{v}}_{3}$) for the extracts obtained from organic and conventional “Golden Delicious” apple varieties (for the three specific time ranges, $\Delta t_{1–3}$: 0–30 s, 30–180 s and 180–900 s). Values are expressed as means (±standard deviation, SD). In a column, values with different superscript letters are significantly different, according to the Tukey HSD (honest significant difference) test (*p* < 0.05). All *p*-level values are presented in the Supplementary Materials (Tables S19–S21).

Code	Organic or Non-Organic Orchard ^1^	DPPH· Reacti on Rateon *t*_1_ Time Range, ${\bar{v}}_{1}\;(\mu M/s)$	DPPH· Reaction Rate on *t*_2_ Time Range, ${\bar{v}}_{2}\;(\mu M/s)$	DPPH· Reaction Rate on *t*_3_ Time Range, ${\bar{v}}_{3}\;(\mu M/s)$
*Gd*(*sh*)*_SRa*	*O*	4.80 (±0.00) ^a^	0.50 (±0.00) ^a^	0.009 (±0.000) ^a^
*Gd*(*co*)*_SRa*	*O*	3.90 (±0.00) ^a^	0.50 (±0.00) ^a^	0.039 (±0.003) ^b^
*Gd*(*sh*)*_SRb*	*O*	5.65 (±0.07) ^a^	0.30 (±0.00) ^a,c^	0.003 (±0.000) ^a^
*Gd*(*co*)*_SRb*	*O*	4.75 (±0.07) ^a^	0.45 (±0.07) ^a^	0.019 (±0.010) ^a,b^
*Gd*(*sh*)*_AR*	*N*	4.25 (±0.21) ^a^	0.60 (±0.00) ^a^	0.013 (±0.000) ^a,b^
*Gd*(*co*)*_AR*	*N*	3.70 (±0.99) ^a,b^	0.70 (±0.14) ^a^	0.017 (±0.013) ^a,b^
*Gd*(*sh*)*_LG*	*N*	5.60 (±0.85) ^a^	0.30 (±0.28) ^a,c^	0.004 (±0.004) ^a^
*Gd*(*co*)*_LG*	*N*	2.10 (±0.00) ^b^	0.85 (±0.07) ^a,b^	0.052 (±0.003) ^b^
*Gd*(*sh*)*_MK*	*N*	1.75 (±0.07) ^b^	0.30 (±0.00) ^a,c^	0.099 (±0.002) ^c^
*Gd*(*co*)*_MK*	*N*	2.05 (±0.64) ^b^	0.13 (±0.11) ^a,c^	0.022 (±0.010) ^a,b^

^1^ *O*—organic orchard; *N*—non-organic orchard; ^a,b,c^ Values with different superscript letters in a column are significantly different, according to the Tukey HSD test (*p* < 0.05).

**Table 6:** Values of the mean DPPH· reaction rates (${\bar{v}}_{1},\;{\bar{v}}_{2}$ and ${\bar{v}}_{3}$) for the extracts obtained from organic and conventional “Florina” apple varieties (for the three specific time ranges, $\Delta t_{1–3}$: 0–30 s, 30–180 s and 180–900 s). Values are expressed as means (±standard deviation, SD). In a column, values with different superscript letters are significantly different, according to the Tukey HSD (honest significant difference) test (*p* < 0.05). All *p*-level values are presented in the Supplementary Materials (Tables S22–S24).

Code	Organic or Non-Organic Orchard ^1^	DPPH· Reacti on Rateon *t*_1_ Time Range, ${\bar{v}}_{1}\;(\mu M/s)$	DPPH· Reaction Rate on *t*_2_ Time Range, ${\bar{v}}_{2}\;(\mu M/s)$	DPPH· Reaction Rate on *t*_3_ Time Range, ${\bar{v}}_{3}\;(\mu M/s)$
*Fl*(*sh*)*_SRa*	*O*	5.60 (±0.00) ^a^	0.30 (±0.00) ^a^	0.002 (±0.000) ^a^
*Fl*(*co*)*_SRa*	*O*	3.85 (±0.07) ^b^	0.65 (±0.07) ^b^	0.016 (±0.003) ^a^
*Fl*(*sh*)*_SRb*	*O*	3.75 (±0.07) ^b^	0.03 (±0.01) ^a,c^	0.001 ^a^
*Fl*(*co*)*_SRb*	*O*	3.75 (±0.07) ^b^	0.10 (±0.00) ^a,c^	0.021 (±0.005) ^a^
*Fl*(*sh*)*_LG*	*N*	6.10 (±0.42) ^a^	0.20 (±0.14) ^a,c^	0.002 (±0.001) ^a^
*Fl*(*co*)*_LG*	*N*	2.75 (±0.07) ^b,c^	0.70 (±0.00) ^b^	0.136 (±0.090) ^a^
*Fl*(*sh*)*_MK*	*N*	3.85 (±0.64) ^b^	0.15 (±0.07) ^a,c^	0.028 (±0.009) ^a^
*Fl*(*co*)*_MK*	*N*	0.95 (±0.21) ^d^	0.09 (±0.01) ^a,c^	0.018 (±0.001) ^a^

^1^ *O*—organic orchard; *N*—non-organic orchard; ^a,b,c,d^ Values with different superscript letters in a column are significantly different, according to the Tukey HSD test (*p* < 0.05).

**Table 7:** Values of the mean DPPH· reaction rates (${\bar{v}}_{1},\;{\bar{v}}_{2}$ and ${\bar{v}}_{3}$) for the extracts obtained from organic and conventional “Generos” apple varieties (for the three specific time ranges, $\Delta t_{1–3}$: 0–30 s, 30–180 s and 180–900 s). Values are expressed as means (±standard deviation, SD). In a column, values with different superscript letters are significantly different, according to the Tukey HSD (honest significant difference) test (*p* < 0.05). All *p*-level values are presented in the Supplementary Materials (Tables S25–S27).

Code	Organic or Non-Organic Orchard ^1^	DPPH· Reaction Rate on *t*_1_ Time Range, ${\bar{v}}_{1}\;(\mu M/s)$	DPPH· Reaction Rate on *t*_2_ Time Range, ${\bar{v}}_{2}\;(\mu M/s)$	DPPH· Reaction Rate on *t*_3_ Time Range, ${\bar{v}}_{3}\;(\mu M/s)$
*Gn*(*sh*)*_SR*	*O*	4.95 (±0.21) ^a^	0.15 (±0.07) ^a^	0.003 (±0.002) ^a^
*Gn*(*co*)*_SR*	*O*	3.95 (±0.21) ^b^	0.40 (±0.00) ^b^	0.066 (±0.001) ^a^
*Gn*(*sh*)*_LG*	*N*	4.65 (±0.07) ^a^	0.40 (±0.00) ^b^	0.015 (±0.002) ^a^
*Gn*(*co*)*_LG*	*N*	2.60 (±0.14) ^c^	0.40 (±0.00) ^b^	0.053 (±0.062) ^a^

^1^ *O*—organic orchard; *N*—non-organic orchard; ^a,b,c^ Values with different superscript letters in a column are significantly different, according to the Tukey HSD test (*p* < 0.05).

**Table 8:** Values of the mean DPPH· reaction rates (${\bar{v}}_{1},\;{\bar{v}}_{2}$ and ${\bar{v}}_{3}$) for the extracts obtained from organic and conventional “Starkrimson” apple varieties (for the three specific time ranges, $\Delta t_{1–3}$: 0–30 s, 30–180 s and 180–900 s). Values are expressed as means (±standard deviation, SD). In a column, values with different superscript letters are significantly different, according to the Tukey HSD (honest significant difference) test (*p* < 0.05). All *p*-level values are presented in the Supplementary Materials (Tables S28–S30).

Code	Organic or Non-Organic Orchard ^1^	DPPH· Reaction Rate on *t*_1_ Time Range, ${\bar{v}}_{1}\;(\mu M/s)$	DPPH· Reaction Rate on *t*_2_ Time Range, ${\bar{v}}_{2}\;(\mu M/s)$	DPPH· Reaction Rate on *t*_3_ Time Range, ${\bar{v}}_{3}\;(\mu M/s)$
*Sk*(*sh*)*_SRa*	*O*	5.90 (±0.14) ^a^	0.20 (±0.00) ^a^	0.002 (±0.001) ^a^
*Sk*(*co*)*_SRa*	*O*	4.30 (±0.00) ^b^	0.45 (±0.07) ^b^	0.023 (±0.004) ^b^
*Sk*(*sh*)_SRb *	*O*	5.90 (±0.42) ^a^	0.04 ^c^	0.001 ^a^
*Sk*(*sh*)*_MK*	*N*	2.70 (±0.14) ^c^	0.50 (±0.00) ^b^	0.074 (±0.004) ^c^
*Sk*(*co*)*_MK*	*N*	1.50 (±0.14) ^d^	0.20 (±0.00) ^a^	0.043 (±0.006) ^d^

^1^ *O*—organic orchard; *N*—non-organic orchard. * One determination for the latest two time ranges; ^a,b,c,d^ Values with different superscript letters in a column are significantly different, according to the Tukey HSD test (*p* < 0.05).

**Table 9:** Codes and description of the organic and conventional apple samples.

Code	Organic or Non-Organic Orchard ^1^	Description
*Gd*(*sh*)*_SRa*	*O*	“Golden Delicious” variety, shell sample “a”, harvested from the organic orchard (Şiria, Arad county, Romania, 46°16′2″ N, 21°38′18″ E)
*Gd*(*co*)*_SRa*	*O*	“Golden Delicious” variety, core sample “a”, harvested from the organic orchard (Şiria, Arad county, Romania, 46°16′2″ N, 21°38′18″ E)
*Gd*(*sh*)*_SRb*	*O*	“Golden Delicious” variety, shell sample “b”, harvested from the organic orchard (Şiria, Arad county, Romania, 46°16′2″ N, 21°38′18″ E)
*Gd*(*co*)*_SRb*	*O*	“Golden Delicious” variety, core sample “b”, harvested from the organic orchard (Şiria, Arad county, Romania, 46°16′2″ N, 21°38′18″ E)
*Gd*(*sh*)*_AR*	*N*	“Golden Delicious” variety, shell sample, harvested directly from the conventional orchard (Arad, Arad county, Romania, 46°10′ N, 21°19′ E)
*Gd*(*co*)*_AR*	*N*	“Golden Delicious” variety, core sample, harvested directly from the conventional orchard (Arad, Arad county, Romania, 46°10′ N, 21°19′ E)
*Gd*(*sh*)*_LG*	*N*	“Golden Delicious” variety, shell sample, harvested from the conventional orchard (Lugoj, Timiş county, Romania, 45°41′10″ N, 21°52′2″ E)
*Gd*(*co*)*_LG*	*N*	“Golden Delicious” variety, core sample, harvested from the conventional orchard (Lugoj, Timiş county, Romania, 45°41′10″ N, 21°52′2″ E)
*Gd*(*sh*)*_MK*	*N*	“Golden Delicious” variety, shell sample, purchased from the supermarket (Timişoara, Timiş county, Romania)
*Gd*(*co*)*_MK*	*N*	“Golden Delicious” variety, core sample, purchased from the supermarket (Timişoara, Timiş county, Romania)
*Fl*(*sh*)*_SRa*	*O*	“Florina” variety, shell sample “a”, harvested from the organic orchard (Şiria, Arad county, Romania, 46°16′2″ N, 21°38′18″ E)
*Fl*(*co*)*_SRa*	*O*	“Florina” variety, core sample “a”, harvested from the organic orchard (Şiria, Arad county, Romania, 46°16′2″ N, 21°38′18″ E)
*Fl*(*sh*)*_SRb*	*O*	“Florina” variety, shell sample “b”, harvested from the organic orchard (Şiria, Arad county, Romania, 46°16′2″ N, 21°38′18″ E)
*Fl*(*co*)*_SRb*	*O*	“Florina” variety, core sample “b”, harvested from the organic orchard (Şiria, Arad county, Romania, 46°16′2″ N, 21°38′18″ E)
*Fl*(*sh*)*_LG*	*N*	“Florina” variety, shell sample, harvested from the conventional orchard (Lugoj, Timiş county, Romania, 45°41′10″ N, 21°52′2″ E)
*Fl*(*co*)*_LG*	*N*	“Florina” variety, core sample, harvested from the conventional orchard (Lugoj, Timiş county, Romania, 45°41′10″ N, 21°52′2″ E)
*Fl*(*sh*)*_MK*	*N*	“Florina” variety, shell sample, purchased from the supermarket (Timişoara, Timiş county, Romania)
*Fl*(*co*)*_MK*	*N*	“Florina” variety, core sample, purchased from the supermarket (Timişoara, Timiş county, Romania)
*Gn*(*sh*)*_SR*	*O*	“Generos” variety, shell sample, harvested from the organic orchard (Şiria, Arad county, Romania, 46°16′2″ N, 21°38′18″ E)
*Gn*(*co*)*_SR*	*O*	“Generos” variety, core sample, harvested from the organic orchard (Şiria, Arad county, Romania, 46°16′2″ N, 21°38′18″ E)
*Gn*(*sh*)*_LG*	*N*	“Generos” variety, shell sample, harvested from the conventional orchard (Lugoj, Timiş county, Romania, 45°41′10″ N, 21°52′2″ E)
*Gn*(*co*)*_LG*	*N*	“Generos” variety, core sample, harvested from the conventional orchard (Lugoj, Timiş county, Romania, 45°41′10″ N, 21°52′2″ E)
*Sk*(*sh*)*_SRa*	*O*	“Starkrimson” variety, shell sample “a”, harvested from the organic orchard (Şiria, Arad county, Romania, 46°16′2″ N, 21°38′18″ E)
*Sk*(*co*)*_SRa*	*O*	“Starkrimson” variety, core sample “a”, harvested from the organic orchard (Şiria, Arad county, Romania, 46°16′2″ N, 21°38′18″ E)
*Sk*(*sh*)*_SRb*	*O*	“Starkrimson” variety, shell sample “b”, harvested from the organic orchard (Şiria, Arad county, Romania, 46°16′2″ N, 21°38′18″ E)
*Sk*(*sh*)*_MK*	*N*	“Starkrimson” variety, shell sample, purchased from the supermarket (Timişoara, Timiş county, Romania)
*Sk*(*co*)*_MK*	*N*	“Starkrimson” variety, core sample, purchased from the supermarket (Timişoara, Timiş county, Romania)

^1^ *O*—organic orchard; *N*—non-organic orchard.

## Data Availability

Not applicable.

## Supplementary Materials

The following are available online at https://www.mdpi.com/article/10.3390/plants10091957/s1, Figure S1: Variation of the radical scavenging activity (*RSA*%) during the spectrophotometric monitoring for the extracts obtained from the “Golden Delicious” apple varieties in the presence of the DPPH· solution: (a) organic shell and core apple extracts, *Gd*(*sh*)*_SRa* and *Gd*(*co*)*_SRa*; (b) organic shell and core apple extracts, *Gd*(*sh*)*_SRb* and *Gd*(*co*)*_SRb*; (c) non-organic shell and core apple extracts, *Gd*(*sh*)*_AR* and *Gd*(*co*)*_AR*; (d) non-organic shell and core apple extracts, *Gd*(*sh*)*_LG* and *Gd*(*co*)*_LG*; (e) non-organic shell and core apple extracts, *Gd*(*sh*)*_MK* and *Gd*(*co*)*_MK* (all determinations are presented as duplicates “*1*” and “*2*”), Figure S2: Variation of the radical scavenging activity (*RSA*%) during the spectrophotometric monitoring for the extracts obtained from the “Florina” apple varieties in the presence of the DPPH· solution: (a) organic shell and core apple extracts, *Fl*(*sh*)*_SRa* and *Fl*(*co*)*_SRa*; (b) organic shell and core apple extracts, *Fl*(*sh*)*_SRb* and *Fl*(*co*)*_SRb*; (c) non-organic shell and core apple extracts, *Fl*(*sh*)*_LG* and *Fl*(*co*)*_LG*; (d) non-organic shell and core apple extracts, *Fl*(*sh*)*_MK* and *Fl*(*co*)*_MK* (all determinations are presented as duplicates “*1*” and “*2*”), Figure S3: Variation of the radical scavenging activity (*RSA*%) during the spectrophotometric monitoring for the extracts obtained from the “Generos” apple varieties in the presence of the DPPH· solution: (a) organic shell and core apple extracts, *Gn*(*sh*)*_SR* and *Gn*(*co*)*_SR*; (b) non-organic shell and core apple extracts, *Gn*(*sh*)*_LG* and *Gn*(*co*)*_LG* (all determinations are presented as duplicates “*1*” and “*2*”), Figure S4: Variation of the radical scavenging activity (*RSA*%) during the spectrophotometric monitoring for the extracts obtained from the “Starkrimson” apple varieties in the presence of the DPPH· solution: (a) organic shell and core apple extracts, *Sk*(*sh*)*_SRa* and *Sk*(*co*)*_SRa*; (b) organic shell apple extracts, *Sk*(*sh*)*_SRb*; (c) non-organic shell and core apple extracts, *Sk*(*sh*)*_MK* and *Sk*(*co*)*_MK* (all determinations are presented as duplicates “*1*” and “*2*”), Table S1: Radical scavenging activity (*RSA*%) at various times of monitoring for the standard antioxidant compound solutions (*Rv_1 mM/0.2 mM/0.1 mM*—resveratrol solutions at concentrations of 1 mM/0.2 m/0.1 mM; *PG_0.2 mM*—propyl gallate solution at a concentration of 0.2 mM). Values are expressed as means (±standard deviation, SD), Table S2: Significance *p*-levels from the Tukey HSD test (honest significant difference) for the antioxidant activity of “Golden Delicious” apple extracts (*RSA*—radical scavenging activity, %, at 1 min; organic shell apple extracts: *Gd*(*sh*)*_SRa* and *Gd*(*sh*)*_SRb*; organic core apple extracts: *Gd*(*co*)*_SRa* and *Gd*(*co*)*_SRb*; non-organic shell apple extracts: *Gd*(*sh*)*_AR*, *Gd*(*sh*)*_LG* and *Gd*(*sh*)*_MK*; non-organic core apple extracts: *Gd*(*co*)*_AR*, *Gd*(*co*)*_LG* and *Gd*(*co*)*_MK*). *p*-level values lower than 0.05 are in bold, Table S3: Significance *p*-levels from the Tukey HSD test (honest significant difference) for the antioxidant activity of “Golden Delicious” apple extracts (*RSA*—radical scavenging activity, %, at 3 min; organic shell apple extracts: *Gd*(*sh*)*_SRa* and *Gd*(*sh*)*_SRb*; organic core apple extracts: *Gd*(*co*)*_SRa* and *Gd*(*co*)*_SRb*; non-organic shell apple extracts: *Gd*(*sh*)*_AR*, *Gd*(*sh*)*_LG* and *Gd*(*sh*)*_MK*; non-organic core apple extracts: *Gd*(*co*)*_AR*, *Gd*(*co*)*_LG* and *Gd*(*co*)*_MK*). *p*-level values lower than 0.05 are in bold, Table S4: Significance *p*-levels from the Tukey HSD test (honest significant difference) for the antioxidant activity of “Golden Delicious” apple extracts (*RSA*—radical scavenging activity, %, at 5 min; organic shell apple extracts: *Gd*(*sh*)*_SRa* and *Gd*(*sh*)*_SRb*; organic core apple extracts: *Gd*(*co*)*_SRa* and *Gd*(*co*)*_SRb*; non-organic shell apple extracts: *Gd*(*sh*)*_AR*, *Gd*(*sh*)*_LG* and *Gd*(*sh*)*_MK*; non-organic core apple extracts: *Gd*(*co*)*_AR*, *Gd*(*co*)*_LG* and *Gd*(*co*)*_MK*). *p*-level values lower than 0.05 are in bold, Table S5: Significance *p*-levels from the Tukey HSD test (honest significant difference) for the antioxidant activity of “Golden Delicious” apple extracts (*RSA*—radical scavenging activity, %, at 15 min; organic shell apple extracts: *Gd*(*sh*)*_SRa* and *Gd*(*sh*)*_SRb*; organic core apple extracts: *Gd*(*co*)*_SRa* and *Gd*(*co*)*_SRb*; non-organic shell apple extracts: *Gd*(*sh*)*_AR*, *Gd*(*sh*)*_LG* and *Gd*(*sh*)*_MK*; non-organic core apple extracts: *Gd*(*co*)*_AR*, *Gd*(*co*)*_LG* and *Gd*(*co*)*_MK*). *p*-level values lower than 0.05 are in bold, Table S6: Significance *p*-levels from the Tukey HSD test (honest significant difference) for the antioxidant activity of “Florina” apple extracts (*RSA*—radical scavenging activity, %, at 1 min; organic shell apple extracts: *Fl*(*sh*)*_SRa* and *Fl*(*sh*)*_SRb*; organic core apple extracts: *Fl*(*co*)*_SRa* and *Fl*(*co*)*_SRb*; non-organic shell apple extracts: *Fl*(*sh*)*_LG* and *Fl*(*sh*)*_MK*; non-organic core apple extracts: *Fl*(*co*)*_LG* and *Fl*(*co*)*_MK*). *p*-level values lower than 0.05 are in bold, Table S7: Significance *p*-levels from the Tukey HSD test (honest significant difference) for the antioxidant activity of “Florina” apple extracts (*RSA*—radical scavenging activity, %, at 3 min; organic shell apple extracts: *Fl*(*sh*)*_SRa* and *Fl*(*sh*)*_SRb*; organic core apple extracts: *Fl*(*co*)*_SRa* and *Fl*(*co*)*_SRb*; non-organic shell apple extracts: *Fl*(*sh*)*_LG* and *Fl*(*sh*)*_MK*; non-organic core apple extracts: *Fl*(*co*)*_LG* and *Fl*(*co*)*_MK*). *p*-level values lower than 0.05 are in bold, Table S8: Significance *p*-levels from the Tukey HSD test (honest significant difference) for the antioxidant activity of “Florina” apple extracts (*RSA*—radical scavenging activity, %, at 5 min; organic shell apple extracts: *Fl*(*sh*)*_SRa* and *Fl*(*sh*)*_SRb*; organic core apple extracts: *Fl*(*co*)*_SRa* and *Fl*(*co*)*_SRb*; non-organic shell apple extracts: *Fl*(*sh*)*_LG* and *Fl*(*sh*)*_MK*; non-organic core apple extracts: *Fl*(*co*)*_LG* and *Fl*(*co*)*_MK*). *p*-level values lower than 0.05 are in bold, Table S9: Significance *p*-levels from the Tukey HSD test (honest significant difference) for the antioxidant activity of “Florina” apple extracts (*RSA*—radical scavenging activity, %, at 15 min; organic shell apple extracts: *Fl*(*sh*)*_SRa* and *Fl*(*sh*)*_SRb*; organic core apple extracts: *Fl*(*co*)*_SRa* and *Fl*(*co*)*_SRb*; non-organic shell apple extracts: *Fl*(*sh*)*_LG* and *Fl*(*sh*)*_MK*; non-organic core apple extracts: *Fl*(*co*)*_LG* and *Fl*(*co*)*_MK*). *p*-level values lower than 0.05 are in bold, Table S10: Significance *p*-levels from the Tukey HSD test (honest significant difference) for the antioxidant activity of “Generos” apple extracts (*RSA*—radical scavenging activity, %, at 1 min; organic shell apple extract: *Gn*(*sh*)*_SR*; organic core apple extract: *Gn*(*co*)*_SR*; non-organic shell apple extract: *Gn*(*sh*)*_LG*; non-organic core apple extract: *Gn*(*co*)*_LG*). *p*-level values lower than 0.05 are in bold, Table S11: Significance *p*-levels from the Tukey HSD test (honest significant difference) for the antioxidant activity of “Generos” apple extracts (*RSA*—radical scavenging activity, %, at 3 min; organic shell apple extract: *Gn*(*sh*)*_SR*; organic core apple extract: *Gn*(*co*)*_SR*; non-organic shell apple extract: *Gn*(*sh*)*_LG*; non-organic core apple extract: *Gn*(*co*)*_LG*). *p*-level values lower than 0.05 are in bold, Table S12: Significance *p*-levels from the Tukey HSD test (honest significant difference) for the antioxidant activity of “Generos” apple extracts (*RSA*—radical scavenging activity, %, at 5 min; organic shell apple extract: *Gn*(*sh*)*_SR*; organic core apple extract: *Gn*(*co*)*_SR*; non-organic shell apple extract: *Gn*(*sh*)*_LG*; non-organic core apple extract: *Gn*(*co*)*_LG*). *p*-level values lower than 0.05 are in bold, Table S13: Significance *p*-levels from the Tukey HSD test (honest significant difference) for the antioxidant activity of “Generos” apple extracts (*RSA*—radical scavenging activity, %, at 15 min; organic shell apple extract: *Gn*(*sh*)*_SR*; organic core apple extract: *Gn*(*co*)*_SR*; non-organic shell apple extract: *Gn*(*sh*)*_LG*; non-organic core apple extract: *Gn*(*co*)*_LG*). *p*-level values lower than 0.05 are in bold, Table S14: Significance *p*-levels from the Tukey HSD test (honest significant difference) for the antioxidant activity of “Starkrimson” apple extracts (*RSA*—radical scavenging activity, %, at 1 min; organic shell apple extracts: *Sk*(*sh*)*_SRa* and *Sk*(*sh*)*_SRb*; organic core apple extract: *Sk*(*co*)*_SRa*; non-organic shell apple extract: *Sk*(*sh*)*_MK*; non-organic core apple extract: *Sk*(*co*)*_LG*). *p*-level values lower than 0.05 are in bold, Table S15: Significance *p*-levels from the Tukey HSD test (honest significant difference) for the antioxidant activity of “Starkrimson” apple extracts (*RSA*—radical scavenging activity, %, at 3 min; organic shell apple extracts: *Sk*(*sh*)*_SRa* and *Sk*(*sh*)*_SRb*; organic core apple extract: *Sk*(*co*)*_SRa*; non-organic shell apple extract: *Sk*(*sh*)*_MK*; non-organic core apple extract: *Sk*(*co*)*_LG*). *p*-level values lower than 0.05 are in bold, Table S16: Significance *p*-levels from the Tukey HSD test (honest significant difference) for the antioxidant activity of “Starkrimson” apple extracts (*RSA*—radical scavenging activity, %, at 5 min; organic shell apple extracts: *Sk*(*sh*)*_SRa* and *Sk*(*sh*)*_SRb*; organic core apple extract: *Sk*(*co*)*_SRa*; non-organic shell apple extract: *Sk*(*sh*)*_MK*; non-organic core apple extract: *Sk*(*co*)*_LG*). *p*-level values lower than 0.05 are in bold, Table S17: Significance *p*-levels from the Tukey HSD test (honest significant difference) for the antioxidant activity of “Starkrimson” apple extracts (*RSA*—radical scavenging activity, %, at 15 min; organic shell apple extracts: *Sk*(*sh*)*_SRa* and *Sk*(*sh*)*_SRb*; organic core apple extract: *Sk*(*co*)*_SRa*; non-organic shell apple extract: *Sk*(*sh*)*_MK*; non-organic core apple extract: *Sk*(*co*)*_LG*). *p*-level values lower than 0.05 are in bold, Figure S5: Variation of the DPPH· concentration during the reaction with antioxidant compounds from the extracts obtained from: (a) organic shell and core “Golden Delicious” apple variety (*Gd*(*sh*)*_SRa* and *Gd*(*co*)*_SRa*); (b) non-organic shell and core “Golden Delicious” apple variety (*Gd*(*sh*)*_LG* and *LG*(*co*)*_AR*); (c) non-organic shell and core “Golden Delicious” apple variety (*Gd*(*sh*)*_MK* and *Gd*(*co*)*_MK*) (all determinations are presented as duplicates “*1*” and “*2*”), Figure S6: Variation of the DPPH· concentration during the reaction with antioxidant compounds from the extracts obtained from: (a) organic shell and core “Florina” apple variety (*Fl*(*sh*)*_SRb* and *Fl*(*co*)*_SRb*); (b) non-organic shell and core “Florina” apple variety (*Fl*(*sh*)*_MK* and *Fl*(*co*)*_MK*); (c) organic shell “Starkrimson” apple variety (*Sk*(*sh*)*_SRb*) (all determinations are presented as duplicates “*1*” and “*2*”), Table S18: Values of the mean DPPH· reaction rates (${\bar{v}}_{1}$, ${\bar{v}}_{2}$ and ${\bar{v}}_{3}$) for the standard antioxidant compound solutions (*Rv_1 mM/0.2 mM/0.1 mM*—resveratrol solutions at concentrations of 1 mM/0.2 m/0.1 mM; *PG_0.2 mM*—propyl gallate solution at a concentration of 0.2 mM). Values are expressed as means (±standard deviation, SD), Table S19: Significance *p*-levels from the Tukey HSD test (honest significant difference) for the DPPH· reaction rate on the Δ*t*_1_ = 0–30 s in the presence of “Golden Delicious” apple extracts (${\bar{v}}_{1}$, μM/s; organic shell apple extracts: *Gd*(*sh*)*_SRa* and *Gd*(*sh*)*_SRb*; organic core apple extracts: *Gd*(*co*)*_SRa* and *Gd*(*co*)*_SRb*; non-organic shell apple extracts: *Gd*(*sh*)*_AR*, *Gd*(*sh*)*_LG* and *Gd*(*sh*)*_MK*; non-organic core apple extracts: *Gd*(*co*)*_AR*, *Gd*(*co*)*_LG* and *Gd*(*co*)*_MK*). *p*-level values lower than 0.05 are in bold, Table S20: Significance *p*-levels from the Tukey HSD test (honest significant difference) for the DPPH· reaction rate on the Δ*t*_2_ = 30–180 s in the presence of “Golden Delicious” apple extracts (${\bar{v}}_{2}$, μM/s; organic shell apple extracts: *Gd*(*sh*)*_SRa* and *Gd*(*sh*)*_SRb*; organic core apple extracts: *Gd*(*co*)*_SRa* and *Gd*(*co*)*_SRb*; non-organic shell apple extracts: *Gd*(*sh*)*_AR*, *Gd*(*sh*)*_LG* and *Gd*(*sh*)*_MK*; non-organic core apple extracts: *Gd*(*co*)*_AR*, *Gd*(*co*)*_LG* and *Gd*(*co*)*_MK*). *p*-level values lower than 0.05 are in bold, Table S21: Significance *p*-levels from the Tukey HSD test (honest significant difference) for the DPPH· reaction rate on the Δ*t*_3_ = 180–900 s in the presence of “Golden Delicious” apple extracts (${\bar{v}}_{3}$, μM/s; organic shell apple extracts: *Gd*(*sh*)*_SRa* and *Gd*(*sh*)*_SRb*; organic core apple extracts: *Gd*(*co*)*_SRa* and *Gd*(*co*)*_SRb*; non-organic shell apple extracts: *Gd*(*sh*)*_AR*, *Gd*(*sh*)*_LG* and *Gd*(*sh*)*_MK*; non-organic core apple extracts: *Gd*(*co*)*_AR*, *Gd*(*co*)*_LG* and *Gd*(*co*)*_MK*). *p*-level values lower than 0.05 are in bold, Table S22: Significance *p*-levels from the Tukey HSD test (honest significant difference) for the DPPH· reaction rate on the Δ*t*_1_ = 0–30 s in the presence of “Florina” apple extracts (${\bar{v}}_{1}$, μM/s; organic shell apple extracts: *Fl*(*sh*)*_SRa* and *Fl*(*sh*)*_SRb*; organic core apple extracts: *Fl*(*co*)*_SRa* and *Fl*(*co*)*_SRb*; non-organic shell apple extracts: *Fl*(*sh*)*_LG* and *Fl*(*sh*)*_MK*; non-organic core apple extracts: *Fl*(*co*)*_LG* and *Fl*(*co*)*_MK*). *p*-level values lower than 0.05 are in bold, Table S23: Significance *p*-levels from the Tukey HSD test (honest significant difference) for the DPPH· reaction rate on the Δ*t*_3_ = 30–180 s in the presence of “Florina” apple extracts (${\bar{v}}_{2}$, μM/s; organic shell apple extracts: *Fl*(*sh*)*_SRa* and *Fl*(*sh*)*_SRb*; organic core apple extracts: *Fl*(*co*)*_SRa* and *Fl*(*co*)*_SRb*; non-organic shell apple extracts: *Fl*(*sh*)*_LG* and *Fl*(*sh*)*_MK*; non-organic core apple extracts: *Fl*(*co*)*_LG* and *Fl*(*co*)*_MK*). *p*-level values lower than 0.05 are in bold, Table S24: Significance *p*-levels from the Tukey HSD test (honest significant difference) for the DPPH· reaction rate on the Δ*t*_3_ = 180–900 s in the presence of “Florina” apple extracts (${\bar{v}}_{3}$, μM/s; organic shell apple extracts: *Fl*(*sh*)*_SRa* and *Fl*(*sh*)*_SRb*; organic core apple extracts: *Fl*(*co*)*_SRa* and *Fl*(*co*)*_SRb*; non-organic shell apple extracts: *Fl*(*sh*)*_LG* and *Fl*(*sh*)*_MK*; non-organic core apple extracts: *Fl*(*co*)*_LG* and *Fl*(*co*)*_MK*). *p*-level values lower than 0.05 are in bold, Table S25: Significance *p*-levels from the Tukey HSD test (honest significant difference) for the DPPH· reaction rate on the Δ*t*_1_ = 0–30 s in the presence of “Generos” apple extracts (${\bar{v}}_{1}$, μM/s; organic shell apple extract: *Gn*(*sh*)*_SR*; organic core apple extract: *Gn*(*co*)*_SR*; non-organic shell apple extract: *Gn*(*sh*)*_LG*; non-organic core apple extract: *Gn*(*co*)*_LG*). *p*-level values lower than 0.05 are in bold, Table S26: Significance *p*-levels from the Tukey HSD test (honest significant difference) for the DPPH· reaction rate on the Δ*t*_2_ = 30–180 s in the presence of “Generos” apple extracts (${\bar{v}}_{2}$, μM/s; organic shell apple extract: *Gn*(*sh*)*_SR*; organic core apple extract: *Gn*(*co*)*_SR*; non-organic shell apple extract: *Gn*(*sh*)*_LG*; non-organic core apple extract: *Gn*(*co*)*_LG*). *p*-level values lower than 0.05 are in bold, Table S27: Significance *p*-levels from the Tukey HSD test (honest significant difference) for the DPPH· reaction rate on the Δ*t*_3_ = 180–900 s in the presence of “Generos” apple extracts (${\bar{v}}_{3}$, μM/s; organic shell apple extract: *Gn*(*sh*)*_SR*; organic core apple extract: *Gn*(*co*)*_SR*; non-organic shell apple extract: *Gn*(*sh*)*_LG*; non-organic core apple extract: *Gn*(*co*)*_LG*). *p*-level values lower than 0.05 are in bold, Table S28: Significance *p*-levels from the Tukey HSD test (honest significant difference) for the DPPH· reaction rate on the Δ*t*_1_ = 0–30 s in the presence of “Starkrimson” apple extracts (${\bar{v}}_{1}$, μM/s; organic shell apple extracts: *Sk*(*sh*)*_SRa* and *Sk*(*sh*)*_SRb*; organic core apple extract: *Sk*(*co*)*_SRa*; non-organic shell apple extract: *Sk*(*sh*)*_MK*; non-organic core apple extract: *Sk*(*co*)*_MK*). *p*-level values lower than 0.05 are in bold, Table S29: Significance *p*-levels from the Tukey HSD test (honest significant difference) for the DPPH· reaction rate on the Δ*t*_2_ = 30–180 s in the presence of “Starkrimson” apple extracts (${\bar{v}}_{2}$, μM/s; organic shell apple extracts: *Sk*(*sh*)*_SRa* and *Sk*(*sh*)*_SRb*; organic core apple extract: *Sk*(*co*)*_SRa*; non-organic shell apple extract: *Sk*(*sh*)*_MK*; non-organic core apple extract: *Sk*(*co*)*_MK*). *p*-level values lower than 0.05 are in bold, Table S30: Significance *p*-levels from the Tukey HSD test (honest significant difference) for the DPPH· reaction rate on the Δ*t*_3_ = 180–900 s in the presence of “Starkrimson” apple extracts (${\bar{v}}_{3}$, μM/s; organic shell apple extracts: *Sk*(*sh*)*_SRa* and *Sk*(*sh*)*_SRb*; organic core apple extract: *Sk*(*co*)*_SRa*; non-organic shell apple extract: *Sk*(*sh*)*_MK*; non-organic core apple extract: *Sk*(*co*)*_MK*). *p*-level values lower than 0.05 are in bold, Figure S7: Correlations between mean DPPH· reaction rate for the first time range of 0–30 s, ${\bar{v}}_{1}$, and antioxidant activity, *RSA*, at various times for all apple extracts. (a) *RSA* at 1 min; (b) *RSA* at 3 min; (c) *RSA* at 5 min; (d) *RSA* at 15 min, Figure S8: Representative dependences between the mean DPPH· reaction rates for the second and third time ranges of 30–180 s and 180–900 s (${\bar{v}}_{2}$ and ${\bar{v}}_{3}$), and antioxidant activity, *RSA*, at various times for all apple extracts. These correlations are not statistically significant. (a) ${\bar{v}}_{2}$ versus *RSA* at 1 min; (b) ${\bar{v}}_{2}$ versus *RSA* at 15 min; (c) ${\bar{v}}_{3}$ versus *RSA* at 1 min, Figure S9: Representative correlations between mean DPPH· reaction rates for the first and third time ranges (0–30 s and 180–900 s, ${\bar{v}}_{1}$ and ${\bar{v}}_{3}$), and antioxidant activity, *RSA*, at various times for all organic apple extracts. (a) ${\bar{v}}_{1}$ versus *RSA* at 1 min; (a) ${\bar{v}}_{3}$ versus *RSA* at 1 min, Equations (S1)–(S4): Linear correlation between antioxidant and DPPH· kinetic parameters and classical analysis parameters for all apple core samples, Equations (S5)–(S9): Linear correlation between antioxidant and DPPH· kinetic parameters and classical analysis parameters for organic apple core samples, Figure S10: PCA results for the antioxidant activity and DPPH· kinetics data of all organic and non-organic apple extracts by variety (“Golden Delicious”—“*Gd*”, orange, “Florina”—“*Fl*”, green, “Generos”—“*Gn*”, turquoise, “Starkrimson”—“*Sk*”, red): (a) *PC*_2_ versus *PC*_1_ scores plot; (b) *PC*_2_ versus *PC*_1_ loadings plot, Figure S11: PCA results for the antioxidant activity and DPPH· kinetics data of all organic and non-organic apple extracts by variety (“Golden Delicious”—“*Gd*”, orange, “Florina”—“*Fl*”, green, “Generos”—“*Gn*”, turquoise, “Starkrimson”—“*Sk*”, red): (a) *PC*_3_ versus *PC*_1_ scores plot; (b) *PC*_3_ versus *PC*_1_ loadings plot; (c) eigenvalues of the correlation matrix, Figure S12: PCA results for the antioxidant activity and DPPH· kinetics data of all organic and non-organic apple extracts by the fruit part (shell—“*S*”, red, and core—“*C*”, blue): (a) *PC*_2_ versus *PC*_1_ scores plot; (b) *PC*_3_ versus *PC*_1_ scores plot, Figure S13: PCA results for the antioxidant activity and DPPH· kinetics data of organic (“*O*”—blue) and non-organic (“*N*”—red) apple extracts: (a) *PC*_2_ versus *PC*_1_ scores plot for the “Golden Delicious” apple variety; (b) (a) *PC*_3_ versus *PC*_1_ scores plot for the “Golden Delicious” apple variety; (c) *PC*_2_ versus *PC*_1_ scores plot for the “Florina” apple variety; (d) *PC*_3_ versus *PC*_1_ scores plot for the “Florina” apple variety; (e) *PC*_2_ versus *PC*_1_ scores plot for the “Generos” apple variety; (f) *PC*_3_ versus *PC*_1_ scores plot for the “Generos” apple variety; (g) *PC*_2_ versus *PC*_1_ scores plot for the “Starkrimson” apple variety; (h) *PC*_3_ versus *PC*_1_ scores plot for the “Starkrimson” apple variety, Figure S14: PCA results for the antioxidant activity and DPPH· kinetics data of organic and non-organic apple extracts: (a) *PC*_2_ versus *PC*_1_ loadings plot for the “Golden Delicious” apple variety; (b) eigenvalues of the correlation matrix for the “Golden Delicious” apple variety; (c) *PC*_2_ versus *PC*_1_ loadings plot for the “Florina” apple variety; (d) eigenvalues of the correlation matrix for the “Florina” apple variety; (e) *PC*_2_ versus *PC*_1_ loadings plot for the “Generos” apple variety; (f) eigenvalues of the correlation matrix for the “Generos” apple variety; (g) *PC*_2_ versus *PC*_1_ loadings plot for the “Starkrimson” apple variety; (h) eigenvalues of the correlation matrix for the “Starkrimson” apple variety.
