# Supplementary material for: Antioxidant Activity and Discrimination of Organic Apples (Malus domestica Borkh.) Cultivated in the Western Region of Romania: A DPPH· Kinetics–PCA Approach
Source: Plants (Basel). 2021 Sep 19;10(9):1957. doi: 10.3390/plants10091957 (PMC8466220; doi:10.3390/plants10091957)
Supplement: Supplementary file 1 [file plants-10-01957-s001.zip › plants-1386777-supplementary-material-New.pdf]

plants

MDPI

Type of the Paper (Article)

Supplementary material

Antioxidant activity and discrimination of organic apples (*Malus domestica* Borkh.) cultivated in the western region of Romania: a DPPH· kinetics – PCA approach

Olimpia Alina Iordănescu [1,¶], Maria Băla [1,¶], Alina Carmen Iuga [1,¶], Dina Gligor (Pane) [2], Ionuţ Dascălu [1,¶], Gabriel Stelian Bujancă [3], Ioan David [4,*], Nicoleta Gabriela Hădărugă [2,4,*], Daniel Ioan Hădărugă [2,5]

[1] Department of Horticulture, Banat's University of Agricultural Sciences and Veterinary Medicine "King Michael I of Romania" from Timişoara, Calea Aradului 119, 300645-Timişoara, Romania; olimpia.iordanescu@yahoo.com (O.A.I.); mariabalamonicabala@yahoo.com (M.B.); i_ali4ever@yahoo.com (A.C.I.); dascalu_ionut91@yahoo.com (I.D.)

[2] Doctoral School "Engineering of Vegetable and Animal Resources", Banat's University of Agricultural Sciences and Veterinary Medicine "King Michael I of Romania" from Timişoara, Calea Aradului 119, 300645-Timişoara, Romania; gligor_dina_bihor@yahoo.com (D.G.P.)

[3] Department of Food Control, Banat's University of Agricultural Sciences and Veterinary Medicine "King Michael I of Romania" from Timişoara, Calea Aradului 119, 300645 Timişoara, Romania; gabrielbujanca@yahoo.com (G.S.B.)

[4] Department of Food Science, Banat's University of Agricultural Sciences and Veterinary Medicine "King Michael I of Romania" from Timişoara, Calea Aradului 119, 300645 –Timişoara, Romania; neda_university@yahoo.com (I.D.); nicoletahadaruga@usab-tm.ro; nico_hadaruga@yahoo.com (N.G.H.)

[5] Department of Applied Chemistry, Organic and Natural Compounds Engineering, Polytechnic University of Timişoara, Carol Telbisz 6, 300001 – Timişoara, Romania; daniel.hadaruga@upt.ro (D.I.H.)

* Correspondence: nico_hadaruga@yahoo.com (N.G.H.); neda_university@yahoo.com (I.D.); Tel.: +40-256-277-423

¶ These authors contributed equally to this work. They are principal authors.

Citation: Iordănescu, O.A.; Băla, M.; Iuga, A.C.; Gligor (Pane), D.; Dascălu, I.; Bujancă, G.S.; David, I.; Hădărugă, N.G.; Hădărugă, D.I. Antioxidant activity and discrimination of organic apples (*Malus domestica* Borkh.) cultivated in the western region of Romania: a DPPH· kinetics – PCA approach . *Plants* **2021**, *10*, 1957. https://doi.org/10.3390/plants10091957

Publisher's Note: MDPI stays neutral with regard to jurisdictional claims in published maps and institutional affiliations.

Copyright: © 2021 by the author. Licensee MDPI, Basel, Switzerland. This article is an open access article distributed under the terms and conditions of the Creative Commons Attribution (CC BY) license (http://creativecommons.org/licenses/by/4.0/).

Abstract: Apple (*Malus domestica* Borkh.) is one of the most used fruit for beverages in Romania. The goal of the study was to evaluate the antioxidant activity and discrimination of various parts of organic and non-organic apple varieties cultivated in the western region of Romania using the DPPH· kinetics – PCA (principal component analysis) approach. Organic and non-organic apples have been subjected to solid-liquid ethanol extraction. Core and shell extracts were mixed with DPPH· and spectrophotometrically monitored at 517 nm. Antioxidant activity and mean DPPH· reaction rate at various time ranges reveals significant differences between organic and non-organic samples, as well as apple parts. Organic core and shell extracts had higher antioxidant activities than the corresponding non-organic samples (74.5-96.9% and 61.9-97.2%, respectively 23.5-94.3% and 59.5-95.5%). Significant differences were observed for the DPPH· reaction rate for the first ½ min, especially in the presence of organic core extracts (3.7-4.8 μM/s). The organic samples were well discriminated by DPPH· kinetics – PCA, the most important variables being the DPPH· reaction rate for the first time range. This is the first DPPH· kinetics – PCA approach applied for discriminating between organic and non-organic fruits and can be useful for evaluating the quality of such type of fruits.

Keywords: apple; *Malus domestica* Borkh.; antioxidant activity; organic and non-organic orchards; DPPH· kinetics; mean DPPH· reaction rate; principal component analysis discrimination

1. Radical scavenging activity of apple extracts

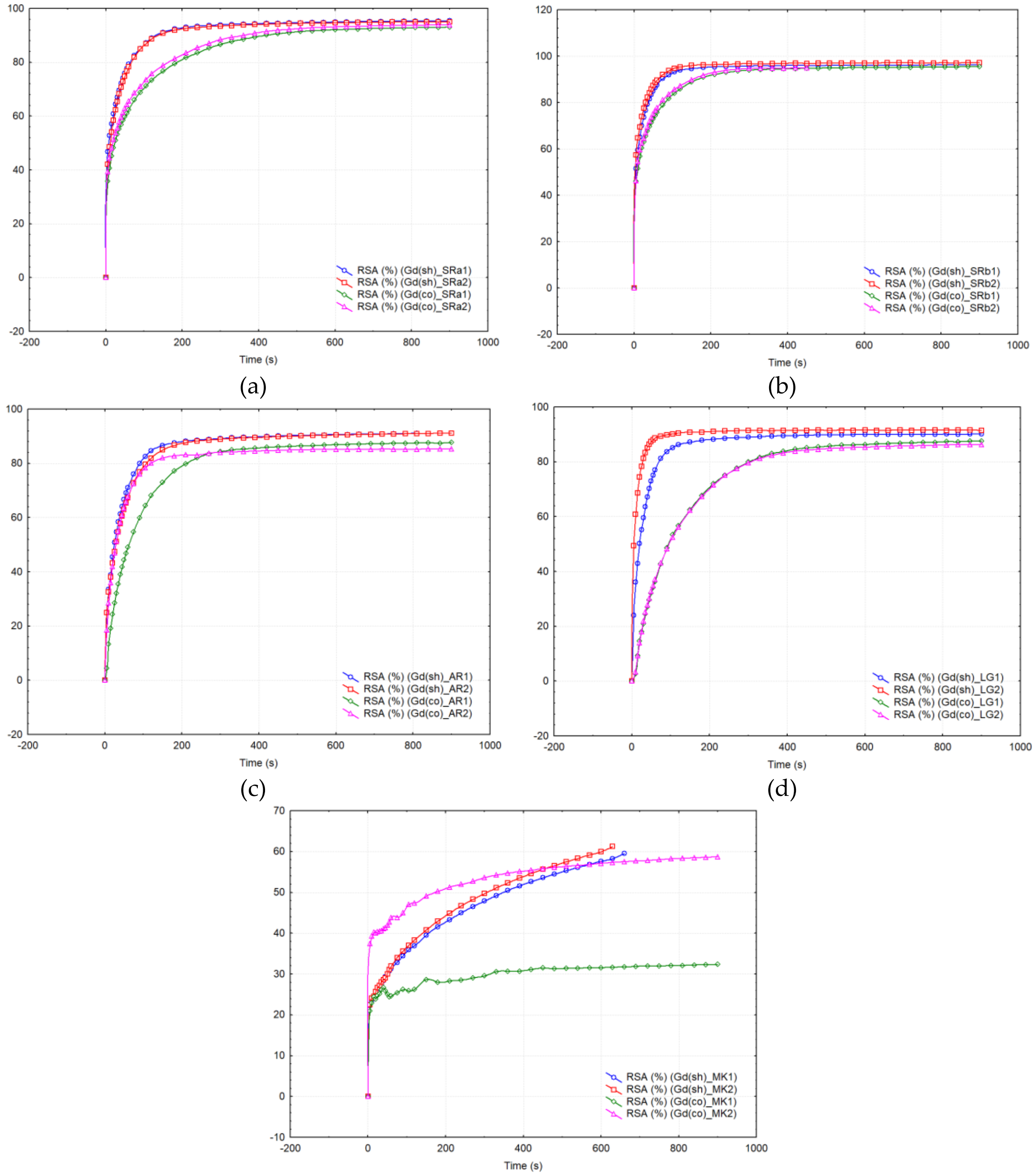

(e)

Figure S1. Variation of the radical scavenging activity (*RSA*, %) during the spectrophotometric monitoring for the extracts obtained from the "Golden Delicious" apple varieties in the presence of the DPPH· solution: (a) organic shell and core apple extracts, *Gd(sh)_SRa* and *Gd(co)_SRa*; (b) organic shell and core apple extracts, *Gd(sh)_SRb* and *Gd(co)_SRb*; (c) non-organic shell and core apple extracts, *Gd(sh)_AR* and *Gd(co)_AR*; (d) non-organic shell and core apple extracts, *Gd(sh)_LG* and *Gd(co)_LG*; (e) non-organic shell and core apple extracts, *Gd(sh)_MK* and *Gd(co)_MK* (all determinations are presented as duplicates "*1*" and "*2*").

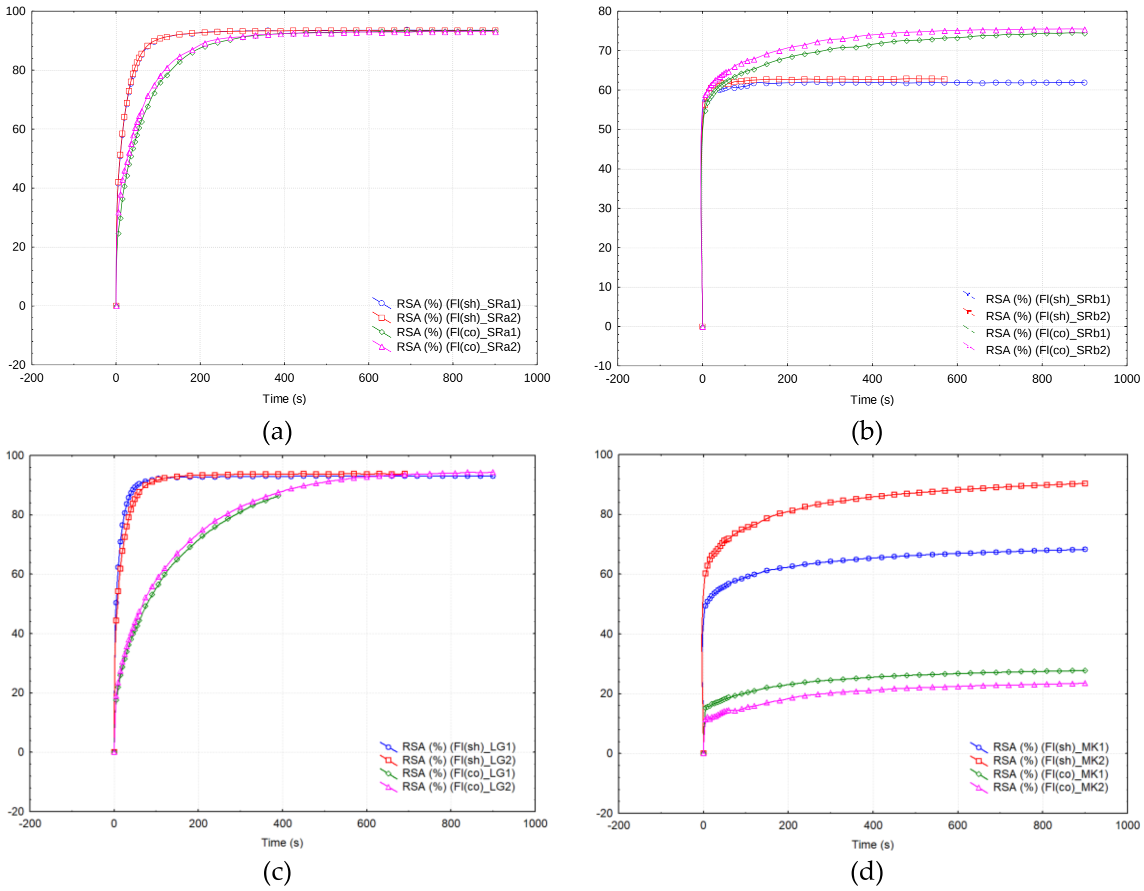

Figure S2. Variation of the radical scavenging activity (*RSA*, %) during the spectrophotometric monitoring for the extracts obtained from the "Florina" apple varieties in the presence of the DPPH· solution: (a) organic shell and core apple extracts, *Fl(sh)_SRa* and *Fl(co)_SRa*; (b) organic shell and core apple extracts, *Fl(sh)_SRb* and *Fl(co)_SRb*; (c) non-organic shell and core apple extracts, *Fl(sh)_LG* and *Fl(co)_LG*; (d) non-organic shell and core apple extracts, *Fl(sh)_MK* and *Fl(co)_MK* (all determinations are presented as duplicates "1" and "2").

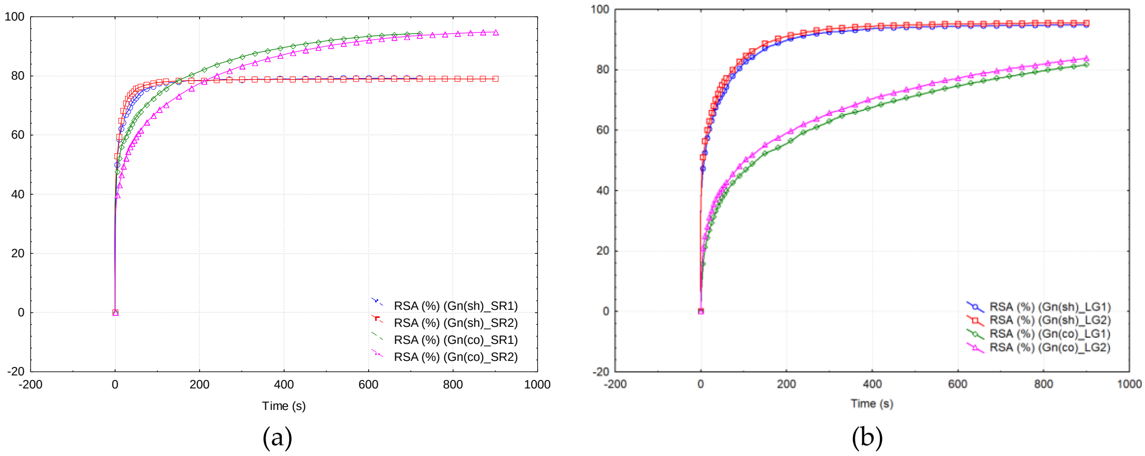

Figure S3. Variation of the radical scavenging activity (*RSA*, %) during the spectrophotometric monitoring for the extracts obtained from the "Generos" apple varieties in the presence of the DPPH· solution: (a) organic shell and core apple extracts, *Gn(sh)_SR* and *Gn(co)_SR*; (b) non-organic shell and core apple extracts, *Gn(sh)_LG* and *Gn(co)_LG* (all determinations are presented as duplicates "1" and "2").

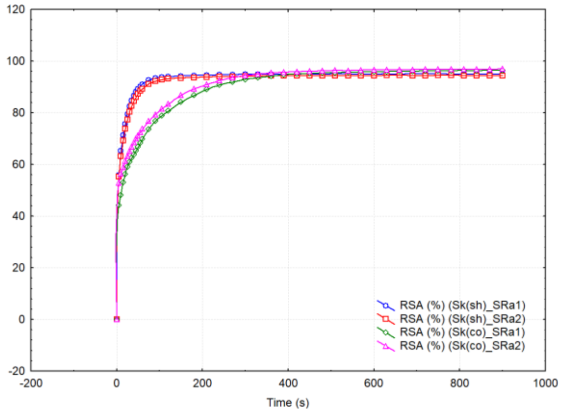

(a)

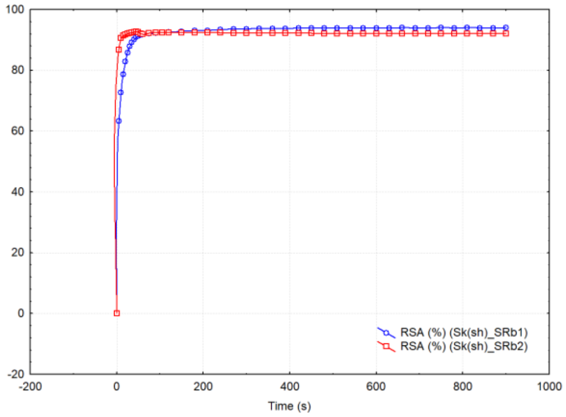

(b)

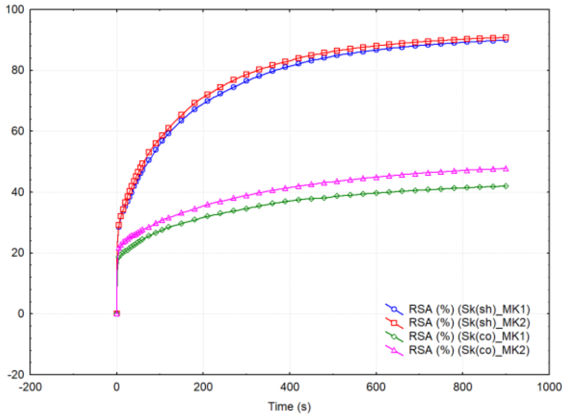

(c)

Figure S4. Variation of the radical scavenging activity (*RSA*, %) during the spectrophotometric monitoring for the extracts obtained from the "Starkrimson" apple varieties in the presence of the DPPH· solution: (a) organic shell and core apple extracts, *Sk(sh)_SRa* and *Sk(co)_SRa*; (b) organic shell apple extracts, *Sk(sh)_SRb*; (c) non-organic shell and core apple extracts, *Sk(sh)_MK* and *Sk(co)_MK* (all determinations are presented as duplicates "*1*" and "*2*").

Table S1. Radical scavenging activity (*RSA*, %) at various times of monitoring for the standard antioxidant compound solutions (*Rv_1mM/0.2mM/0.1mM* – resveratrol solutions at concentrations of 1 mM/0.2 m/0.1 mM; *PG_0.2mM* – propyl gallate solution at a concentration of 0.2 mM). Values are expressed as mean (± standard deviation, SD).

Code	Antioxidant type	*RSA* (1 min) (%)	*RSA* (3 min) (%)	*RSA* (5 min) (%)	*RSA* (15 min) (%)
Rv_1mM	*Natural*	57.97 (±0.13)	68.11 (±0.24)	72.99 (±0.41)	84.17 (±0.62)
Rv_0.2mM	*Natural*	48.89 (±0.25)	52.86 (±0.22)	54.86 (±0.17)	59.55 (±0.03)
Rv_0.1mM *	*Natural*	45.22	47.53	48.71	51.41
PG_0.2mM *	*Synthetic*	67.96	73.74	76.76	82.46

* Single determination.

Table S2. Significance *p*-levels from the Tukey HSD test (honestly significant difference) for the antioxidant activity of "Golden Delicious" apple extracts (*RSA* – radical scavenging activity, %, at 1 min; organic shell apple extracts: *Gd(sh)_SRa* and *Gd(sh)_SRb*; organic core apple extracts: *Gd(co)_SRa* and *Gd(co)_SRb*; non-organic shell apple extracts: *Gd(sh)_AR*, *Gd(sh)_LG* and *Gd(sh)_MK*; non-organic core apple extracts: *Gd(co)_AR*, *Gd(co)_LG* and *Gd(co)_MK*). *p*-level values lower than 0.05 are bolded.

	Gd(sh)_SRa	*Gd(co)_SRa*	*Gd(sh)_SRb*	*Gd(co)_SRb*	*Gd(sh)_AR*	*Gd(co)_AR*	*Gd(sh)_LG*	*Gd(co)_LG*	*Gd(sh)_MK*	*Gd(co)_MK*
Gd(sh)_SRa		0.499545	0.872778	0.999999	0.894093	0.179092	0.999591	**0.002226**	**0.000985**	**0.001460**
Gd(co)_SRa			0.072473	0.666544	0.996926	0.995563	0.242760	**0.042211**	**0.014432**	**0.024694**
Gd(sh)_SRb				0.728343	0.219464	**0.021963**	0.994094	**0.000535**	**0.000351**	**0.000422**
Gd(co)_SRb					0.971651	0.270224	0.991501	**0.003220**	**0.001352**	**0.002065**
Gd(sh)_AR						0.807790	0.598859	**0.013759**	**0.004997**	**0.008255**
Gd(co)_AR							0.076196	0.138944	**0.047085**	0.081613
Gd(sh)_LG								**0.001145**	**0.000580**	**0.000799**
Gd(co)_LG									0.997776	0.999992
Gd(sh)_MK										0.999991
Gd(co)_MK										

Table S3. Significance *p*-levels from the Tukey HSD test (honestly significant difference) for the antioxidant activity of "Golden Delicious" apple extracts (*RSA* – radical scavenging activity, %, at 3 min; organic shell apple extracts: *Gd(sh)_SRa* and *Gd(sh)_SRb*; organic core apple extracts: *Gd(co)_SRa* and *Gd(co)_SRb*; non-organic shell apple extracts: *Gd(sh)_AR*, *Gd(sh)_LG* and *Gd(sh)_MK*; non-organic core apple extracts: *Gd(co)_AR*, *Gd(co)_LG* and *Gd(co)_MK*). *p*-level values lower than 0.05 are bolded.

	Gd(sh)_SRa	*Gd(co)_SRa*	*Gd(sh)_SRb*	*Gd(co)_SRb*	*Gd(sh)_AR*	*Gd(co)_AR*	*Gd(sh)_LG*	*Gd(co)_LG*	*Gd(sh)_MK*	*Gd(co)_MK*
Gd(sh)_SRa		0.485095	0.999075	1.000000	0.988950	0.443705	0.999861	**0.017177**	**0.000264**	**0.000247**
Gd(co)_SRa			0.213973	0.562604	0.935137	1.000000	0.767941	0.380467	**0.000781**	**0.000488**
Gd(sh)_SRb				0.996029	0.807238	0.191421	0.954288	**0.006860**	**0.000238**	**0.000231**
Gd(co)_SRb					0.996497	0.518305	0.999991	**0.021111**	**0.000270**	**0.000250**
Gd(sh)_AR						0.910963	0.999978	0.066925	**0.000343**	**0.000281**
Gd(co)_AR							0.725307	0.418624	**0.000844**	**0.000516**
Gd(sh)_LG								**0.036423**	**0.000294**	**0.000261**
Gd(co)_LG									**0.014365**	**0.006346**
Gd(sh)_MK										0.999600
Gd(co)_MK										

Table S4. Significance *p*-levels from the Tukey HSD test (honestly significant difference) for the antioxidant activity of "Golden Delicious" apple extracts (*RSA* – radical scavenging activity, %, at 5 min; organic shell apple extracts: *Gd(sh)_SRa* and *Gd(sh)_SRb*; organic core apple extracts: *Gd(co)_SRa* and *Gd(co)_SRb*; non-organic shell apple extracts: *Gd(sh)_AR*, *Gd(sh)_LG* and *Gd(sh)_MK*; non-organic core apple extracts: *Gd(co)_AR*, *Gd(co)_LG* and *Gd(co)_MK*). *p*-level values lower than 0.05 are bolded.

	Gd(sh)_SRa	*Gd(co)_SRa*	*Gd(sh)_SRb*	*Gd(co)_SRb*	*Gd(sh)_AR*	*Gd(co)_AR*	*Gd(sh)_LG*	*Gd(co)_LG*	*Gd(sh)_MK*	*Gd(co)_MK*
Gd(sh)_SRa		0.970322	0.999951	1.000000	0.994870	0.753758	0.999401	0.344341	**0.000406**	**0.000264**
Gd(co)_SRa			0.827140	0.948120	1.000000	0.999519	0.999939	0.892232	**0.000939**	**0.000368**
Gd(sh)_SRb				0.999996	0.925854	0.503100	0.972232	0.188302	**0.000331**	**0.000249**
Gd(co)_SRb					0.987889	0.693010	0.997888	0.298677	**0.000383**	**0.000259**
Gd(sh)_AR						0.993306	1.000000	0.777670	**0.000737**	**0.000331**
Gd(co)_AR							0.973012	0.996578	**0.001790**	**0.000524**
Gd(sh)_LG								0.665226	**0.000620**	**0.000309**
Gd(co)_LG									**0.004718**	**0.001043**
Gd(sh)_MK										0.926547
Gd(co)_MK										

Table S5. Significance *p*-levels from the Tukey HSD test (honestly significant difference) for the antioxidant activity of "Golden Delicious" apple extracts (*RSA* – radical scavenging activity, %, at 15 min; organic shell apple extracts: *Gd(sh)_SRa* and *Gd(sh)_SRb*; organic core apple extracts: *Gd(co)_SRa* and *Gd(co)_SRb*; non-organic shell apple extracts: *Gd(sh)_AR*, *Gd(sh)_LG* and *Gd(sh)_MK*; non-organic core apple extracts: *Gd(co)_AR*, *Gd(co)_LG* and *Gd(co)_MK*). *p*-level values lower than 0.05 are bolded.

	Gd(sh)_SRa	*Gd(co)_SRa*	*Gd(sh)_SRb*	*Gd(co)_SRb*	*Gd(sh)_AR*	*Gd(co)_AR*	*Gd(sh)_LG*	*Gd(co)_LG*	*Gd(sh)_MK*	*Gd(co)_MK*
Gd(sh)_SRa		0.999999	1.000000	1.000000	0.998909	0.878171	0.997991	0.901594	**0.003771**	**0.000382**
Gd(co)_SRa			0.999841	0.999999	0.999990	0.963572	0.999968	0.974090	**0.005554**	**0.000450**
Gd(sh)_SRb				1.000000	0.991074	0.774181	0.986621	0.805789	**0.002828**	**0.000345**
Gd(co)_SRb					0.999001	0.881186	0.998144	0.904286	**0.003809**	**0.000383**
Gd(sh)_AR						0.997442	1.000000	0.998667	**0.009242**	**0.000588**
Gd(co)_AR							0.998572	1.000000	**0.027036**	**0.001171**
Gd(sh)_LG								0.999311	**0.010004**	**0.000615**
Gd(co)_LG									**0.024646**	**0.001097**
Gd(sh)_MK										0.368525
Gd(co)_MK										

Table S6. Significance *p*-levels from the Tukey HSD test (honestly significant difference) for the antioxidant activity of "Florina" apple extracts (*RSA* – radical scavenging activity, %, at 1 min; organic shell apple extracts: *Fl(sh)_SRa* and *Fl(sh)_SRb*; organic core apple extracts: *Fl(co)_SRa* and *Fl(co)_SRb*; non-organic shell apple extracts: *Fl(sh)_LG* and *Fl(sh)_MK*; non-organic core apple extracts: *Fl(co)_LG* and *Fl(co)_MK*). *p*-level values lower than 0.05 are bolded.

	Fl(sh)_SRa	*Fl(co)_SRa*	*Fl(sh)_SRb*	*Fl(co)_SRb*	*Fl(sh)_LG*	*Fl(co)_LG*	*Fl(sh)_MK*	*Fl(co)_MK*
Fl(sh)_SRa		**0.027098**	**0.039373**	**0.022688**	0.983522	**0.000823**	**0.026934**	**0.000240**
Fl(co)_SRa			0.999054	1.000000	**0.011265**	**0.048304**	1.000000	**0.000381**
Fl(sh)_SRb				0.999863	**0.018646**	0.214254	0.999105	**0.001194**
Fl(co)_SRb					**0.009581**	0.058291	1.000000	**0.000398**
Fl(sh)_LG						**0.000544**	**0.011203**	**0.000239**
Fl(co)_LG							**0.048612**	**0.004340**
Fl(sh)_MK								**0.000381**
Fl(co)_MK								

Table S7. Significance *p*-levels from the Tukey HSD test (honestly significant difference) for the antioxidant activity of "Florina" apple extracts (*RSA* – radical scavenging activity, %, at 3 min; organic shell apple extracts: *Fl(sh)_SRa* and *Fl(sh)_SRb*; organic core apple extracts: *Fl(co)_SRa* and *Fl(co)_SRb*; non-organic shell apple extracts: *Fl(sh)_LG* and *Fl(sh)_MK*; non-organic core apple extracts: *Fl(co)_LG* and *Fl(co)_MK*). *p*-level values lower than 0.05 are bolded.

	Fl(sh)_SRa	*Fl(co)_SRa*	*Fl(sh)_SRb*	*Fl(co)_SRb*	*Fl(sh)_LG*	*Fl(co)_LG*	*Fl(sh)_MK*	*Fl(co)_MK*
Fl(sh)_SRa		0.922382	**0.026205**	**0.030315**	1.000000	**0.040164**	**0.049479**	**0.000245**
Fl(co)_SRa			0.076107	0.114097	0.890435	0.153669	0.190584	**0.000276**
Fl(sh)_SRb				0.965683	**0.024010**	0.916234	0.863625	**0.003643**
Fl(co)_SRb					**0.027200**	0.999992	0.999678	**0.000600**
Fl(sh)_LG						**0.035951**	**0.044213**	**0.000244**
Fl(co)_LG							0.999999	**0.000538**
Fl(sh)_MK								**0.000493**
Fl(co)_MK								

Table S8. Significance *p*-levels from the Tukey HSD test (honestly significant difference) for the antioxidant activity of "Florina" apple extracts (*RSA* – radical scavenging activity, %, at 5 min; organic shell apple extracts: *Fl(sh)_SRa* and *Fl(sh)_SRb*; organic core apple extracts: *Fl(co)_SRa* and *Fl(co)_SRb*; non-organic shell apple extracts: *Fl(sh)_LG* and *Fl(sh)_MK*; non-organic core apple extracts: *Fl(co)_LG* and *Fl(co)_MK*). *p*-level values lower than 0.05 are bolded.

	Fl(sh)_SRa	*Fl(co)_SRa*	*Fl(sh)_SRb*	*Fl(co)_SRb*	*Fl(sh)_LG*	*Fl(co)_LG*	*Fl(sh)_MK*	*Fl(co)_MK*
Fl(sh)_SRa		0.999901	**0.030882**	0.059906	1.000000	0.500284	0.103185	**0.000275**
Fl(co)_SRa			**0.043073**	0.091623	0.999887	0.683938	0.158556	**0.000281**
Fl(sh)_SRb				0.868982	**0.030671**	0.218954	0.693211	**0.006921**
Fl(co)_SRb					0.059381	0.598877	0.999512	**0.000756**
Fl(sh)_LG						0.496658	0.102264	**0.000275**
Fl(co)_LG							0.828447	**0.000364**
Fl(sh)_MK								**0.000597**
Fl(co)_MK								

Table S9. Significance *p*-levels from the Tukey HSD test (honestly significant difference) for the antioxidant activity of "Florina" apple extracts (*RSA* – radical scavenging activity, %, at 15 min; organic shell apple extracts: *Fl(sh)_SRa* and *Fl(sh)_SRb*; organic core apple extracts: *Fl(co)_SRa* and *Fl(co)_SRb*; non-organic shell apple extracts: *Fl(sh)_LG* and *Fl(sh)_MK*; non-organic core apple extracts: *Fl(co)_LG* and *Fl(co)_MK*). *p*-level values lower than 0.05 are bolded.

	Fl(sh)_SRa	*Fl(co)_SRa*	*Fl(sh)_SRb*	*Fl(co)_SRb*	*Fl(sh)_LG*	*Fl(co)_LG*	*Fl(sh)_MK*	*Fl(co)_MK*
Fl(sh)_SRa		1.000000	0.059998	0.199107	1.000000	0.999781	0.424263	**0.000372**
Fl(co)_SRa			0.062027	0.207429	1.000000	0.999878	0.439695	**0.000375**
Fl(sh)_SRb				0.758658	0.059828	0.088732	0.479451	**0.022706**
Fl(co)_SRb					0.198413	0.317788	0.994823	**0.001532**
Fl(sh)_LG						0.999770	0.422965	**0.000372**
Fl(co)_LG							0.622299	**0.000417**
Fl(sh)_MK								**0.000977**
Fl(co)_MK								

Table S10. Significance *p*-levels from the Tukey HSD test (honestly significant difference) for the antioxidant activity of "Generos" apple extracts (*RSA* – radical scavenging activity, %, at 1 min; organic shell apple extract: *Gn(sh)_SR*; organic core apple extract: *Gn(co)_SR*; non-organic shell apple extract: *Gn(sh)_LG*; non-organic core apple extract: *Gn(co)_LG*). *p*-level values lower than 0.05 are bolded.

	Gn(sh)_SR	*Gn(co)_SR*	*Gn(sh)_LG*	*Gn(co)_LG*
Gn(sh)_SR		0.064085	0.997978	**0.001100**
Gn(co)_SR			0.055782	**0.003931**
Gn(sh)_LG				**0.001055**
Gn(co)_LG				

Table S11. Significance *p*-levels from the Tukey HSD test (honestly significant difference) for the antioxidant activity of "Generos" apple extracts (*RSA* – radical scavenging activity, %, at 3 min; organic shell apple extract: *Gn(sh)_SR*; organic core apple extract: *Gn(co)_SR*; non-organic shell apple extract: *Gn(sh)_LG*; non-organic core apple extract: *Gn(co)_LG*). *p*-level values lower than 0.05 are bolded.

	Gn(sh)_SR	*Gn(co)_SR*	*Gn(sh)_LG*	*Gn(co)_LG*
Gn(sh)_SR		0.998480	**0.017927**	**0.001438**
Gn(co)_SR			**0.016259**	**0.001505**
Gn(sh)_LG				**0.000472**
Gn(co)_LG				

Table S12. Significance *p*-levels from the Tukey HSD test (honestly significant difference) for the antioxidant activity of "Generos" apple extracts (*RSA* – radical scavenging activity, %, at 5 min; organic shell apple extract: *Gn(sh)_SR*; organic core apple extract: *Gn(co)_SR*; non-organic shell apple extract: *Gn(sh)_LG*; non-organic core apple extract: *Gn(co)_LG*). *p*-level values lower than 0.05 are bolded.

	Gn(sh)_SR	*Gn(co)_SR*	*Gn(sh)_LG*	*Gn(co)_LG*
Gn(sh)_SR		**0.047922**	**0.002524**	**0.002490**
Gn(co)_SR			**0.019332**	**0.000786**
Gn(sh)_LG				**0.000386**
Gn(co)_LG				

Table S13. Significance *p*-levels from the Tukey HSD test (honestly significant difference) for the antioxidant activity of "Generos" apple extracts (*RSA* – radical scavenging activity, %, at 15 min; organic shell apple extract: *Gn(sh)_SR*; organic core apple extract: *Gn(co)_SR*; non-organic shell apple extract: *Gn(sh)_LG*; non-organic core apple extract: *Gn(co)_LG*). *p*-level values lower than 0.05 are bolded.

	Gn(sh)_SR	*Gn(co)_SR*	*Gn(sh)_LG*	*Gn(co)_LG*
Gn(sh)_SR		**0.000387**	**0.000374**	**0.036497**
Gn(co)_SR			0.922697	**0.000654**
Gn(sh)_LG				**0.000593**
Gn(co)_LG				

Table S14. Significance *p*-levels from the Tukey HSD test (honestly significant difference) for the antioxidant activity of "Starkrimson" apple extracts (*RSA* – radical scavenging activity, %, at 1 min; organic shell apple extracts: *Sk(sh)_SRa* and *Sk(sh)_SRb*; organic core apple extract: *Sk(co)_SRa*; non-organic shell apple extract: *Sk(sh)_MK*; non-organic core apple extract: *Sk(co)_LG*). *p*-level values lower than 0.05 are bolded.

	Sk(sh)_SRa	*Sk(co)_SRa*	*Sk(sh)_SRb*	*Sk(sh)_MK*	*Sk(co)_MK*
Sk(sh)_SRa		**0.001069**	0.890317	**0.000272**	**0.000271**
Sk(co)_SRa			**0.000784**	**0.000475**	**0.000271**
Sk(sh)_SRb				**0.000271**	**0.000271**
Sk(sh)_MK					**0.000548**
Sk(co)_MK					

Table S15. Significance *p*-levels from the Tukey HSD test (honestly significant difference) for the antioxidant activity of "Starkrimson" apple extracts (*RSA* – radical scavenging activity, %, at 3 min; organic shell apple extracts: *Sk(sh)_SRa* and *Sk(sh)_SRb*; organic core apple extract: *Sk(co)_SRa*; non-organic shell apple extract: *Sk(sh)_MK*; non-organic core apple extract: *Sk(co)_LG*). *p*-level values lower than 0.05 are bolded.

	Sk(sh)_SRa	*Sk(co)_SRa*	*Sk(sh)_SRb*	*Sk(sh)_MK*	*Sk(co)_MK*
Sk(sh)_SRa		0.056558	0.902596	**0.000300**	**0.000271**
Sk(co)_SRa			0.131536	**0.000499**	**0.000271**
Sk(sh)_SRb				**0.000316**	**0.000271**
Sk(sh)_MK					**0.000272**
Sk(co)_MK					

Table S16. Significance *p*-levels from the Tukey HSD test (honestly significant difference) for the antioxidant activity of "Starkrimson" apple extracts (*RSA* – radical scavenging activity, %, at 5 min; organic shell apple extracts: *Sk(sh)_SRa* and *Sk(sh)_SRb*; organic core apple extract: *Sk(co)_SRa*; non-organic shell apple extract: *Sk(sh)_MK*; non-organic core apple extract: *Sk(co)_LG*). *p*-level values lower than 0.05 are bolded.

	Sk(sh)_SRa	*Sk(co)_SRa*	*Sk(sh)_SRb*	*Sk(sh)_MK*	*Sk(co)_MK*
Sk(sh)_SRa		0.982634	0.856420	**0.001030**	**0.000271**
Sk(co)_SRa			0.987631	**0.001261**	**0.000271**
Sk(sh)_SRb				**0.001553**	**0.000271**
Sk(sh)_MK					**0.000271**
Sk(co)_MK					

Table S17. Significance *p*-levels from the Tukey HSD test (honestly significant difference) for the antioxidant activity of "Starkrimson" apple extracts (*RSA* – radical scavenging activity, %, at 15 min; organic shell apple extracts: *Sk(sh)_SRa* and *Sk(sh)_SRb*; organic core apple extract: *Sk(co)_SRa*; non-organic shell apple extract: *Sk(sh)_MK*; non-organic core apple extract: *Sk(co)_LG*). *p*-level values lower than 0.05 are bolded.

	Sk(sh)_SRa	*Sk(co)_SRa*	*Sk(sh)_SRb*	*Sk(sh)_MK*	*Sk(co)_MK*
Sk(sh)_SRa		0.862829	0.901720	0.321557	**0.000271**
Sk(co)_SRa			0.455069	0.119220	**0.000271**
Sk(sh)_SRb				0.698331	**0.000271**
Sk(sh)_MK					**0.000272**
Sk(co)_MK					

2. DPPH· kinetics approach for the apple extracts

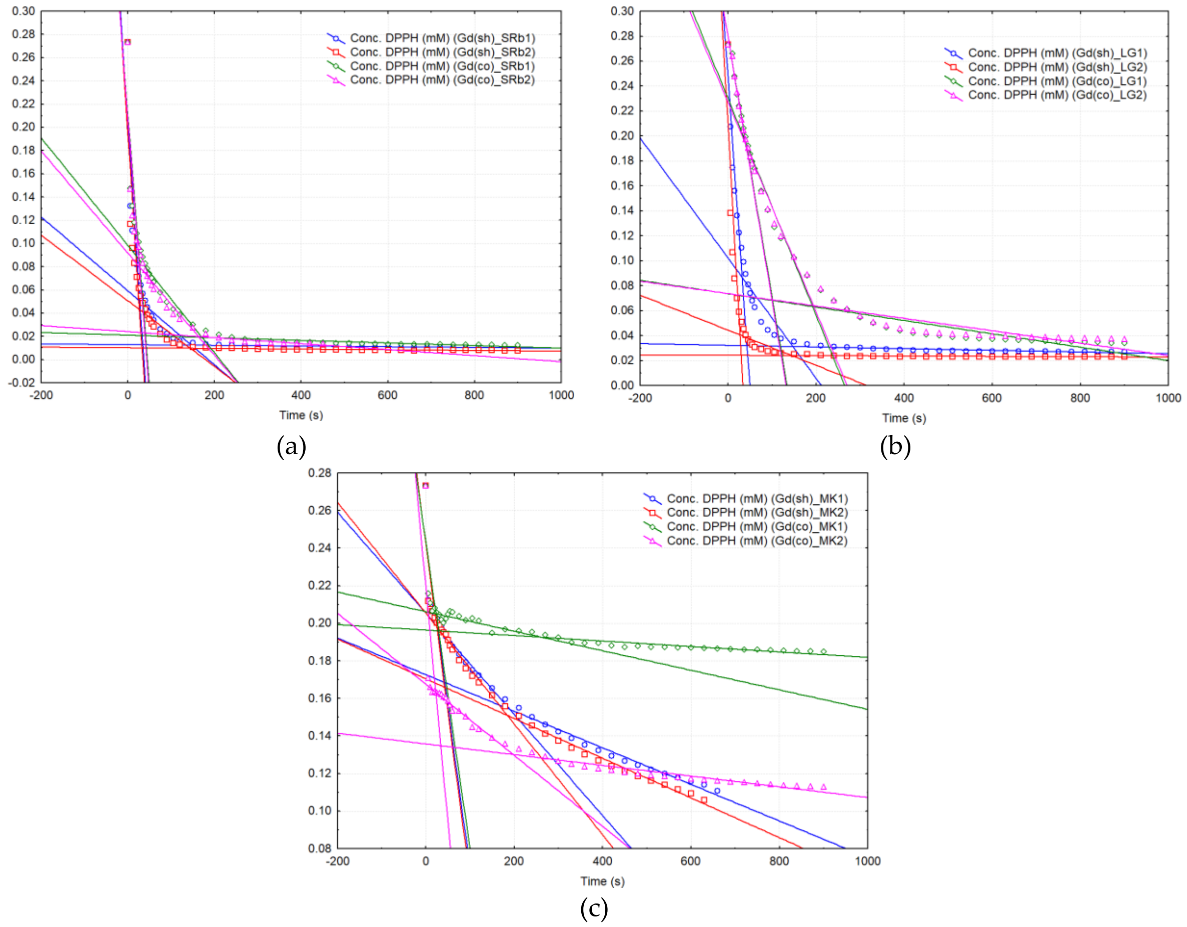

Figure S5. Variation of the DPPH· concentration during the reaction with antioxidant compounds from the extracts obtained from: (a) organic shell and core "Golden Delicious" apple variety (*Gd(sh)_SRa* and *Gd(co)_SRa*); (b) non-organic shell and core "Golden Delicious" apple variety (*Gd(sh)_LG* and *LG(co)_AR*); (c) non-organic shell and core "Golden Delicious" apple variety (*Gd(sh)_MK* and *Gd(co)_MK*) (all determinations are presented as duplicates "*1*" and "*2*").

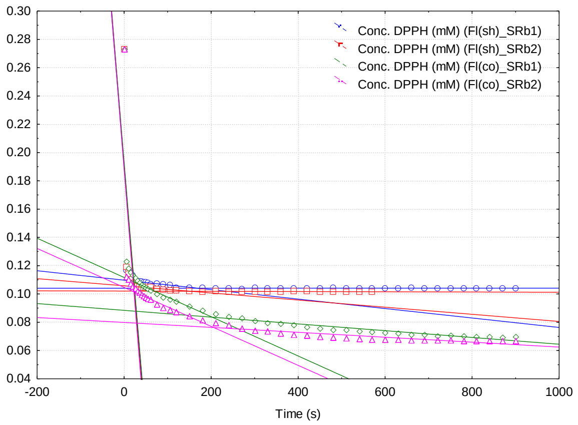

(a)

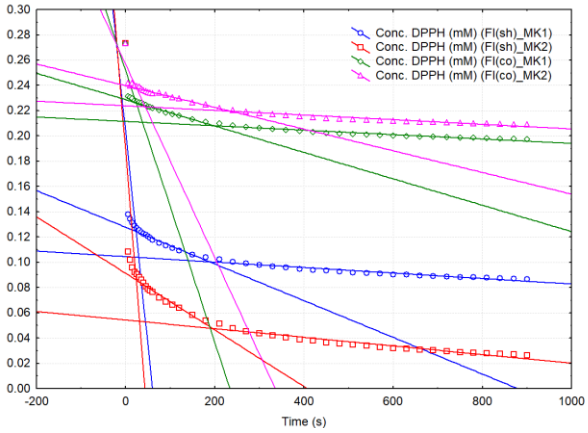

(b)

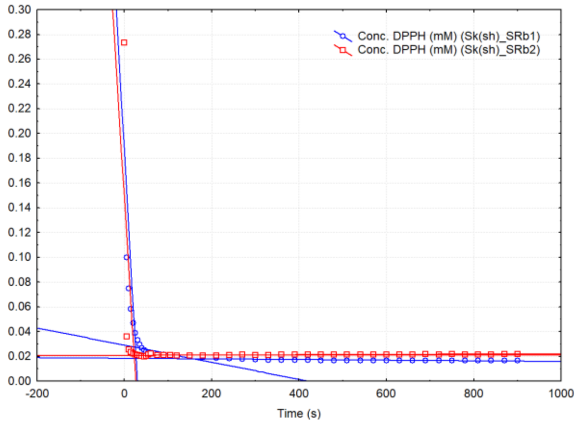

(c)

Figure S6. Variation of the DPPH· concentration during the reaction with antioxidant compounds from the extracts obtained from: (a) organic shell and core "Florina" apple variety (*Fl(sh)_SRb* and *Fl(co)_SRb*); (b) non-organic shell and core "Florina" apple variety (*Fl(sh)_MK* and *Fl(co)_MK*); (c) organic shell "Starkrimson" apple variety (*Sk(sh)_SRb*) (all determinations are presented as duplicates "1" and "2").

Table S18. Values of the mean DPPH· reaction rates ($\bar{v}_1$, $\bar{v}_2$ and $\bar{v}_3$) for the standard antioxidant compound solutions (*Rv_1mM/0.2mM/0.1mM* – resveratrol solutions at concentrations of 1 mM/0.2 m/0.1 mM; *PG_0.2mM* – propyl gallate solution at a concentration of 0.2 mM). Values are expressed as mean (± standard deviation, SD).

**Code**	**Antioxidant type**	**DPPH· reaction rate on t_1 time range, $\bar{v}_1$ (μM/s)**	**DPPH· reaction rate on t_2 time range, $\bar{v}_2$ (μM/s)**	**DPPH· reaction rate on t_3 time range, $\bar{v}_3$ (μM/s)**
Rv_1mM	*Natural*	3.40 (±0.00)	0.30 (±0.00)	0.057 (±0.001)
Rv_0.2mM	*Natural*	2.85 (±0.07)	0.10 (±0.00)	0.024 (±0.001)
*Rv_0.1mM**	*Natural*	2.60	0.06	0.014
*PG_0.2mM**	*Synthetic*	4.20	0.20	0.030

* Single determination.

Table S19. Significance *p*-levels from the Tukey HSD test (honestly significant difference) for the DPPH· reaction rate on the Δt_1 = 0-30 s in the presence of "Golden Delicious" apple extracts ($\bar{v}_1$, μM/s; organic shell apple extracts: *Gd(sh)_SRa* and *Gd(sh)_SRb*; organic core apple extracts: *Gd(co)_SRa* and *Gd(co)_SRb*; non-organic shell apple extracts: *Gd(sh)_AR*, *Gd(sh)_LG* and *Gd(sh)_MK*; non-organic core apple extracts: *Gd(co)_AR*, *Gd(co)_LG* and *Gd(co)_MK*). *p*-level values lower than 0.05 are bolded.

	Gd(sh)_SRa	*Gd(co)_SRa*	*Gd(sh)_SRb*	*Gd(co)_SRb*	*Gd(sh)_AR*	*Gd(co)_AR*	*Gd(sh)_LG*	*Gd(co)_LG*	*Gd(sh)_MK*	*Gd(co)_MK*
Gd(sh)_SRa		0.652404	0.710204	1.000000	0.959733	0.427526	0.765769	**0.004040**	**0.001660**	**0.003535**
Gd(co)_SRa			0.066461	0.710204	0.997956	0.999976	0.077501	0.056972	**0.019549**	**0.048841**
Gd(sh)_SRb				0.652404	0.191007	**0.035915**	1.000000	**0.000601**	**0.000376**	**0.000551**
Gd(co)_SRb					0.977003	0.480397	0.710204	**0.004624**	**0.001875**	**0.004040**
Gd(sh)_AR						0.959733	0.220521	**0.019549**	**0.007017**	**0.016821**
Gd(co)_AR							**0.041878**	0.105214	**0.035915**	0.090333
Gd(sh)_LG								**0.000653**	**0.000397**	**0.000601**
Gd(co)_LG									0.997956	1.000000
Gd(sh)_MK										0.999363
Gd(co)_MK										

Table S20. Significance *p*-levels from the Tukey HSD test (honestly significant difference) for the DPPH· reaction rate on the Δt_2 = 30-180 s in the presence of "Golden Delicious" apple extracts ($\bar{v}_2$, μM/s; organic shell apple extracts: *Gd(sh)_SRa* and *Gd(sh)_SRb*; organic core apple extracts: *Gd(co)_SRa* and *Gd(co)_SRb*; non-organic shell apple extracts: *Gd(sh)_AR*, *Gd(sh)_LG* and *Gd(sh)_MK*; non-organic core apple extracts: *Gd(co)_AR*, *Gd(co)_LG* and *Gd(co)_MK*). *p*-level values lower than 0.05 are bolded.

	Gd(sh)_SRa	*Gd(co)_SRa*	*Gd(sh)_SRb*	*Gd(co)_SRb*	*Gd(sh)_AR*	*Gd(co)_AR*	*Gd(sh)_LG*	*Gd(co)_LG*	*Gd(sh)_MK*	*Gd(co)_MK*
Gd(sh)_SRa		1.000000	0.714566	0.999962	0.992149	0.714566	0.714566	0.150658	0.714566	0.111020
Gd(co)_SRa			0.714566	0.999962	0.992149	0.714566	0.714566	0.150658	0.714566	0.111020
Gd(sh)_SRb				0.913919	0.276348	0.079347	1.000000	**0.011555**	1.000000	0.830878
Gd(co)_SRb					0.913919	0.472044	0.913919	0.079347	0.913919	0.207817
Gd(sh)_AR						0.992149	0.276348	0.472044	0.276348	**0.030312**
Gd(co)_AR							0.079347	0.913919	0.079347	**0.008606**
Gd(sh)_LG								**0.011555**	1.000000	0.830878
Gd(co)_LG									**0.011555**	**0.001610**
Gd(sh)_MK										0.830878
Gd(co)_MK										

Table S21. Significance *p*-levels from the Tukey HSD test (honestly significant difference) for the DPPH· reaction rate on the Δt_3 = 180-900 s in the presence of "Golden Delicious" apple extracts ($\bar{v}_3$, μM/s; organic shell apple extracts: *Gd(sh)_SRa* and *Gd(sh)_SRb*; organic core apple extracts: *Gd(co)_SRa* and *Gd(co)_SRb*; non-organic shell apple extracts: *Gd(sh)_AR*, *Gd(sh)_LG* and *Gd(sh)_MK*; non-organic core apple extracts: *Gd(co)_AR*, *Gd(co)_LG* and *Gd(co)_MK*). *p*-level values lower than 0.05 are bolded.

	Gd(sh)_SRa	*Gd(co)_SRa*	*Gd(sh)_SRb*	*Gd(co)_SRb*	*Gd(sh)_AR*	*Gd(co)_AR*	*Gd(sh)_LG*	*Gd(co)_LG*	*Gd(sh)_MK*	*Gd(co)_MK*
Gd(sh)_SRa		**0.017361**	0.984368	0.879938	0.999653	0.946999	0.995897	**0.001429**	**0.000225**	0.655124
Gd(co)_SRa			**0.004507**	0.138460	**0.040365**	0.101565	**0.005686**	0.623085	**0.000268**	0.259677
Gd(sh)_SRb				0.377600	0.819871	0.482708	1.000000	**0.000565**	**0.000225**	0.209722
Gd(co)_SRb					0.994389	1.000000	0.463340	**0.008468**	**0.000225**	0.999953
Gd(sh)_AR						0.999357	0.893729	**0.002814**	**0.000225**	0.923374
Gd(co)_AR							0.578600	**0.006327**	**0.000225**	0.998962
Gd(sh)_LG								**0.000654**	**0.000225**	0.266166
Gd(co)_LG									**0.000690**	**0.015990**
Gd(sh)_MK										**0.000226**
Gd(co)_MK										

Table S22. Significance *p*-levels from the Tukey HSD test (honestly significant difference) for the DPPH· reaction rate on the Δt_1 = 0-30 s in the presence of "Florina" apple extracts ($\bar{v}_1$, μM/s; organic shell apple extracts: *Fl(sh)_SRa* and *Fl(sh)_SRb*; organic core apple extracts: *Fl(co)_SRa* and *Fl(co)_SRb*; non-organic shell apple extracts: *Fl(sh)_LG* and *Fl(sh)_MK*; non-organic core apple extracts: *Fl(co)_LG* and *Fl(co)_MK*). *p*-level values lower than 0.05 are bolded.

	Fl(sh)_SRa	*Fl(co)_SRa*	*Fl(sh)_SRb*	*Fl(co)_SRb*	*Fl(sh)_LG*	*Fl(co)_LG*	*Fl(sh)_MK*	*Fl(co)_MK*
Fl(sh)_SRa		**0.008713**	**0.022322**	**0.006380**	0.716882	**0.000608**	**0.008713**	**0.000240**
Fl(co)_SRa			1.000000	0.999950	**0.002081**	0.088110	1.000000	**0.000566**
Fl(sh)_SRb				1.000000	**0.005858**	0.224680	1.000000	**0.001717**
Fl(co)_SRb					**0.001625**	0.129777	0.999950	**0.000657**
Fl(sh)_LG						**0.000349**	**0.002081**	**0.000238**
Fl(co)_LG							0.088110	**0.007442**
Fl(sh)_MK								**0.000566**
Fl(co)_MK								

Table S23. Significance *p*-levels from the Tukey HSD test (honestly significant difference) for the DPPH· reaction rate on the Δt_3 = 30-180 s in the presence of "Florina" apple extracts ($\bar{v}_2$, μM/s; organic shell apple extracts: *Fl(sh)_SRa* and *Fl(sh)_SRb*; organic core apple extracts: *Fl(co)_SRa* and *Fl(co)_SRb*; non-organic shell apple extracts: *Fl(sh)_LG* and *Fl(sh)_MK*; non-organic core apple extracts: *Fl(co)_LG* and *Fl(co)_MK*). *p*-level values lower than 0.05 are bolded.

	Fl(sh)_SRa	*Fl(co)_SRa*	*Fl(sh)_SRb*	*Fl(co)_SRb*	*Fl(sh)_LG*	*Fl(co)_LG*	*Fl(sh)_MK*	*Fl(co)_MK*
Fl(sh)_SRa		**0.013312**	0.111716	0.172921	0.776893	**0.006349**	0.405884	0.152349
Fl(co)_SRa			**0.001582**	**0.001052**	**0.003231**	0.990386	**0.001773**	**0.000984**
Fl(sh)_SRb				0.971984	0.454293	**0.001042**	0.762975	0.983318
Fl(co)_SRb					0.776893	**0.000682**	0.990386	1.000000
Fl(sh)_LG						**0.001773**	0.990386	0.723953
Fl(co)_LG							**0.001052**	**0.000647**
Fl(sh)_MK								0.980223
Fl(co)_MK								

Table S24. Significance *p*-levels from the Tukey HSD test (honestly significant difference) for the DPPH· reaction rate on the Δt_3 = 180-900 s in the presence of "Florina" apple extracts ($\bar{v}_3$, μM/s; organic shell apple extracts: *Fl(sh)_SRa* and *Fl(sh)_SRb*; organic core apple extracts: *Fl(co)_SRa* and *Fl(co)_SRb*; non-organic shell apple extracts: *Fl(sh)_LG* and *Fl(sh)_MK*; non-organic core apple extracts: *Fl(co)_LG* and *Fl(co)_MK*). *p*-level values lower than 0.05 are bolded.

	Fl(sh)_SRa	*Fl(co)_SRa*	*Fl(sh)_SRb*	*Fl(co)_SRb*	*Fl(sh)_LG*	*Fl(co)_LG*	*Fl(sh)_MK*	*Fl(co)_MK*
Fl(sh)_SRa		0.999826	1.000000	0.998759	1.000000	0.062393	0.991061	0.999570
Fl(co)_SRa			0.999915	1.000000	0.999791	0.099255	0.999919	1.000000
Fl(sh)_SRb				0.999463	1.000000	0.139493	0.996299	0.999803
Fl(co)_SRb					0.998593	0.117372	0.999998	1.000000
Fl(sh)_LG						0.061604	0.990302	0.999498
Fl(co)_LG							0.150630	0.106424
Fl(sh)_MK								0.999976
Fl(co)_MK								

Table S25. Significance *p*-levels from the Tukey HSD test (honestly significant difference) for the DPPH· reaction rate on the Δt_1 = 0-30 s in the presence of "Generos" apple extracts ($\bar{v}_1$, μM/s; organic shell apple extract: *Gn(sh)_SR*; organic core apple extract: *Gn(co)_SR*; non-organic shell apple extract: *Gn(sh)_LG*; non-organic core apple extract: *Gn(co)_LG*). *p*-level values lower than 0.05 are bolded.

	Gn(sh)_SR	*Gn(co)_SR*	*Gn(sh)_LG*	*Gn(co)_LG*
Gn(sh)_SR		**0.014238**	0.401220	**0.000763**
Gn(co)_SR			**0.047895**	**0.004838**
Gn(sh)_LG				**0.001129**
Gn(co)_LG				

Table S26. Significance *p*-levels from the Tukey HSD test (honestly significant difference) for the DPPH· reaction rate on the Δt_2 = 30-180 s in the presence of "Generos" apple extracts ($\bar{v}_2$, μM/s; organic shell apple extract: *Gn(sh)_SR*; organic core apple extract: *Gn(co)_SR*; non-organic shell apple extract: *Gn(sh)_LG*; non-organic core apple extract: *Gn(co)_LG*). *p*-level values lower than 0.05 are bolded.

	Gn(sh)_SR	*Gn(co)_SR*	*Gn(sh)_LG*	*Gn(co)_LG*
Gn(sh)_SR		**0.007443**	**0.007443**	**0.007443**
Gn(co)_SR			1.000000	1.000000
Gn(sh)_LG				1.000000
Gn(co)_LG				

Table S27. Significance *p*-levels from the Tukey HSD test (honestly significant difference) for the DPPH· reaction rate on the Δt_3 = 180-900 s in the presence of "Generos" apple extracts ($\bar{v}_3$, μM/s; organic shell apple extract: *Gn(sh)_SR*; organic core apple extract: *Gn(co)_SR*; non-organic shell apple extract: *Gn(sh)_LG*; non-organic core apple extract: *Gn(co)_LG*). *p*-level values lower than 0.05 are bolded.

	Gn(sh)_SR	*Gn(co)_SR*	*Gn(sh)_LG*	*Gn(co)_LG*
Gn(sh)_SR		0.307240	0.974877	0.458049
Gn(co)_SR			0.450686	0.971691
Gn(sh)_LG				0.647153
Gn(co)_LG				

Table S28. Significance *p*-levels from the Tukey HSD test (honestly significant difference) for the DPPH· reaction rate on the Δt_1 = 0-30 s in the presence of "Starkrimson" apple extracts ($\bar{v}_1$, μM/s; organic shell apple extracts: *Sk(sh)_SRa* and *Sk(sh)_SRb*; organic core apple extract: *Sk(co)_SRa*; non-organic shell apple extract: *Sk(sh)_MK*; non-organic core apple extract: *Sk(co)_MK*). *p*-level values lower than 0.05 are bolded.

	Sk(sh)_SRa	*Sk(co)_SRa*	*Sk(sh)_SRb*	*Sk(sh)_MK*	*Sk(co)_MK*
Sk(sh)_SRa		**0.004159**	1.000000	**0.000356**	**0.000275**
Sk(co)_SRa			**0.004159**	**0.004159**	**0.000476**
Sk(sh)_SRb				**0.000356**	**0.000275**
Sk(sh)_MK					**0.014434**
Sk(co)_MK					

Table S29. Significance *p*-levels from the Tukey HSD test (honestly significant difference) for the DPPH· reaction rate on the Δt_2 = 30-180 s in the presence of "Starkrimson" apple extracts ($\bar{v}_2$, μM/s; organic shell apple extracts: *Sk(sh)_SRa* and *Sk(sh)_SRb*; organic core apple extract: *Sk(co)_SRa*; non-organic shell apple extract: *Sk(sh)_MK*; non-organic core apple extract: *Sk(co)_MK*). *p*-level values lower than 0.05 are bolded.

	Sk(sh)_SRa	*Sk(co)_SRa*	*Sk(sh)_SRb*	*Sk(sh)_MK*	*Sk(co)_MK*
Sk(sh)_SRa		**0.006906**	**0.038351**	**0.003110**	1.000000
Sk(co)_SRa			**0.000816**	0.703256	**0.006906**
Sk(sh)_SRb				**0.000558**	**0.038351**
Sk(sh)_MK					**0.003110**
Sk(co)_MK					

Table S30. Significance *p*-levels from the Tukey HSD test (honestly significant difference) for the DPPH· reaction rate on the Δt_3 = 180-900 s in the presence of "Starkrimson" apple extracts ($\bar{v}_3$, μM/s; organic shell apple extracts: *Sk(sh)_SRa* and *Sk(sh)_SRb*; organic core apple extract: *Sk(co)_SRa*; non-organic shell apple extract: *Sk(sh)_MK*; non-organic core apple extract: *Sk(co)_MK*). *p*-level values lower than 0.05 are bolded.

	Sk(sh)_SRa	*Sk(co)_SRa*	*Sk(sh)_SRb*	*Sk(sh)_MK*	*Sk(co)_MK*
Sk(sh)_SRa		**0.012772**	0.999924	**0.000277**	**0.000735**
Sk(co)_SRa			**0.011632**	**0.000397**	**0.014135**
Sk(sh)_SRb				**0.000276**	**0.000698**
Sk(sh)_MK					**0.002299**
Sk(co)_MK					

3. Correlations and principal component analysis (PCA) of the antioxidant activity and DPPH· kinetics of apple extracts

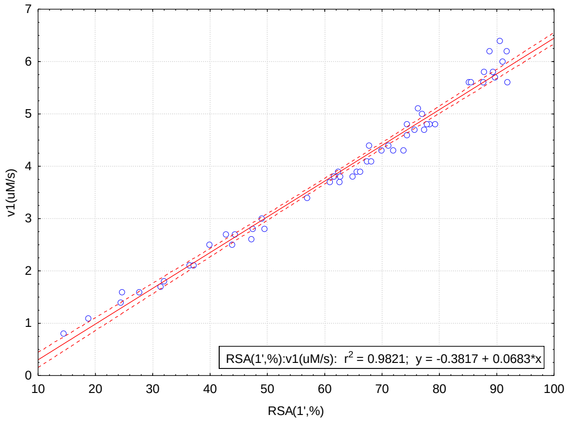

$$\bar{v}_1 = -0.38(\pm 0.08) + 0.068(\pm 0.001) \cdot RSA(1')$$

$n = 54$, $r^2 = 0.982$, $F = 2855$, $s = 0.20$, $p < 0.00001$

(a)

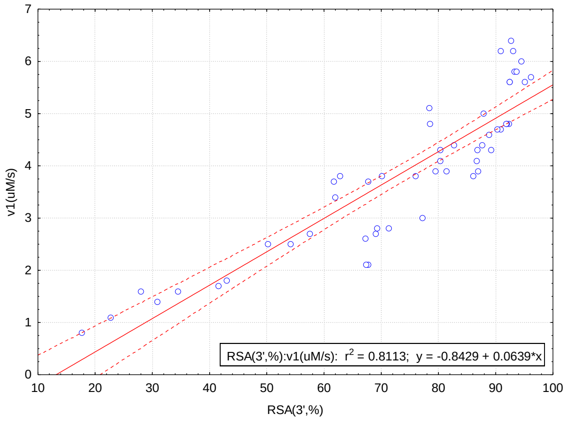

$$\bar{v}_1 = -0.84(\pm 0.33) + 0.064(\pm 0.004) \cdot RSA(3')$$

$n = 54$, $r^2 = 0.901$, $F = 224$, $s = 0.64$, $p < 0.00001$

(b)

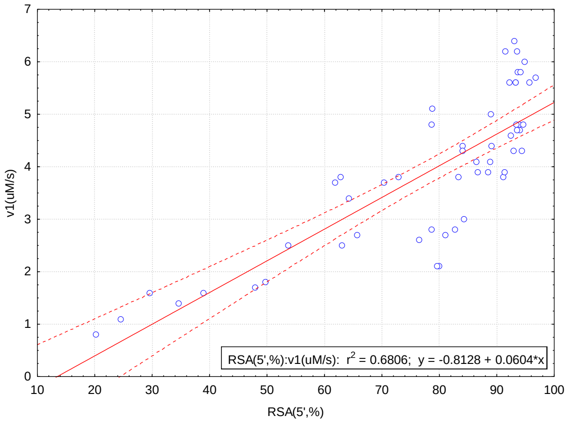

$$\bar{v}_1 = -0.81(\pm 0.46) + 0.060(\pm 0.006) \cdot RSA(5')$$

$n = 54$, $r^2 = 0.825$, $F = 111$, $s = 0.84$, $p < 0.00001$

(c)

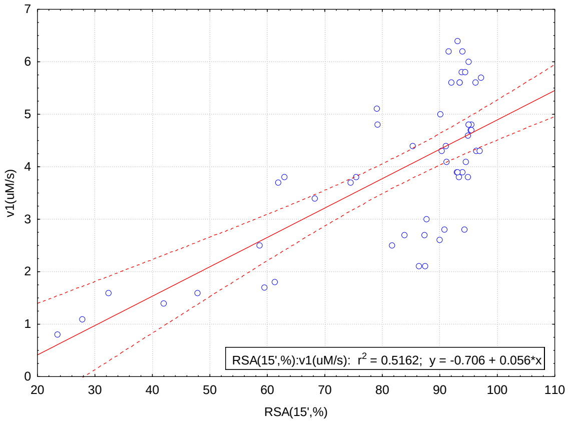

$$\bar{v}_1 = -0.71(\pm 0.64) + 0.056(\pm 0.008) \cdot RSA(15')$$

$n = 54$, $r^2 = 0.718$, $F = 55$, $s = 1.03$, $p < 0.00001$

(d)

Figure S7. Correlations between mean DPPH· reaction rate for the first time range of 0-30 s, $\bar{v}_1$, and antioxidant activity, *RSA*, at various times for all apple extracts. (a) *RSA* at 1 min; (b) *RSA* at 3 min; (c) *RSA* at 5 min; (d) *RSA* at 15 min.

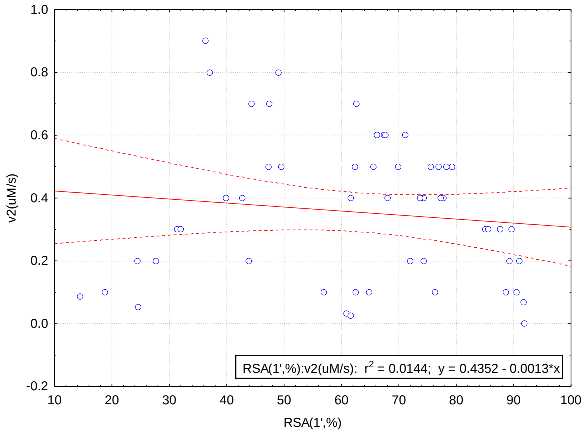

(a)

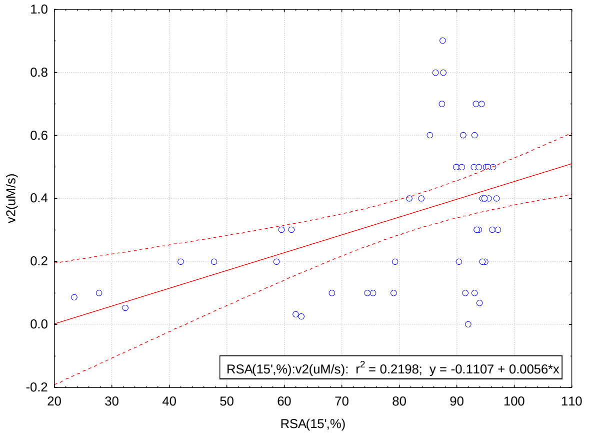

(b)

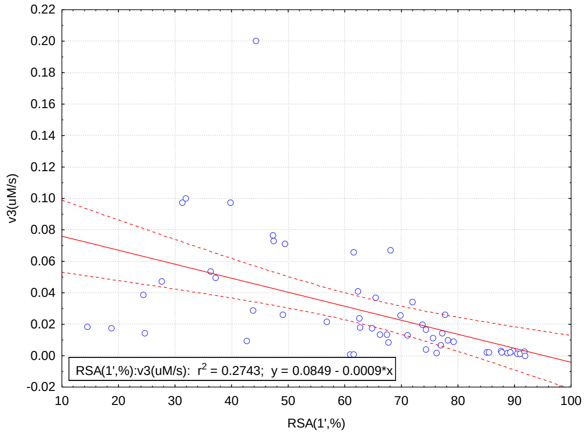

(c)

Figure S8. Representative dependences between the mean DPPH· reaction rates for the second and third time ranges of 30-180 s and 180-900 s ($\bar{v}_2$ and $\bar{v}_3$), and antioxidant activity, *RSA*, at various times for all apple extracts. These correlations are not statistically significant. (a) $\bar{v}_2$ *vs. RSA* at 1 min; (b) $\bar{v}_2$ *vs. RSA* at 15 min; (c) $\bar{v}_3$ *vs. RSA* at 1 min.

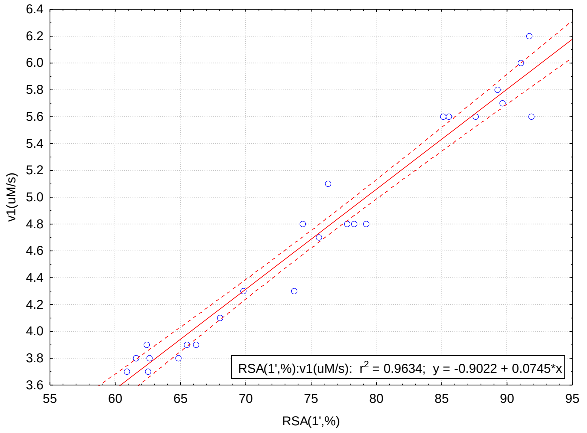

$$\bar{v}_1 = -0.90(\pm 0.22) + 0.074(\pm 0.003) \cdot RSA(1')$$

$n = 26$, $r^2 = 0.982$, $F = 632$, $s = 0.16$, $p < 0.00001$

(a)

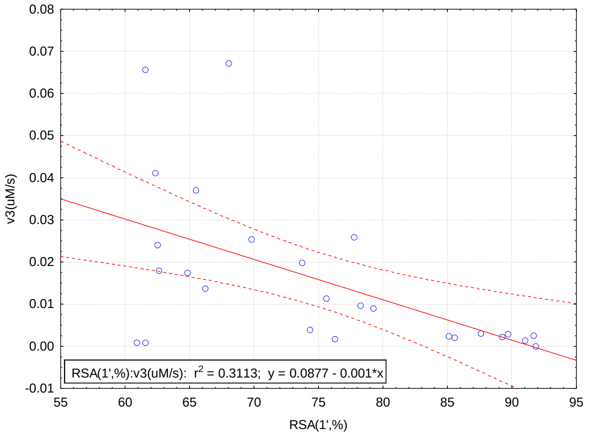

Not statistically significant

(b)

Figure S9. Representative correlations between mean DPPH· reaction rates for the first and third time ranges (0-30 s and 180-900 s, $\bar{v}_1$ and $\bar{v}_3$), and antioxidant activity, *RSA*, at various times for all organic apple extracts. (a) $\bar{v}_1$ *vs. RSA* at 1 min; (a) $\bar{v}_3$ *vs. RSA* at 1 min.

Linear correlation between antioxidant and DPPH· kinetic parameters and classical analysis parameters for all apple core samples (Eqs. S1-S4)

$$RSA(3') = -103.5(\pm 88.8) + 31.02(\pm 16.83) \cdot pH \quad \text{(S1)}$$

$n = 26$, $r = 0.352$, $F = 3.4$, $s = 25.6$, $p < 0.08$

$$RSA(5') = -112.5(\pm 88.9) + 33.62(\pm 16.85) \cdot pH \quad \text{(S2)}$$

$n = 26$, $r = 0.377$, $F = 4.0$, $s = 25.6$, $p < 0.06$

$$RSA(15') = -112.3(\pm 83.4) + 34.84(\pm 15.81) \cdot pH \quad \text{(S3)}$$

$n = 26$, $r = 0.410$, $F = 4.9$, $s = 24.05$, $p < 0.04$

$$\bar{v}_1 = -1.65(\pm 0.68) + 0.38(\pm 0.13) \cdot pH \quad \text{(S4)}$$

$n = 26$, $r = 0.516$, $F = 8.7$, $s = 0.20$, $p < 0.007$

Linear correlation between antioxidant and DPPH· kinetic parameters and classical analysis parameters for organic apple core samples (Eqs. S5-S9)

$$RSA(1') = 21.78(\pm 7.77) + 3.40(\pm 0.60) \cdot Sugar\_content \quad \text{(S5)}$$

$n = 12$, $r = 0.880$, $F = 34.5$, $s = 2.93$, $p < 0.0002$

$$RSA(3') = 16.97 + 4.81(\pm 1.42) \cdot Sugar\_content \quad \text{(S6)}$$

$n = 12$, $r = 0.732$, $F = 11.5$, $s = 7.2$, $p < 0.007$

$$RSA(5') = 15.69 + 5.25(\pm 1.70) \cdot Sugar\_content \quad \text{(S7)}$$

$n = 12$, $r = 0.698$, $F = 9.5$, $s = 8.6$, $p < 0.01$

$$RSA(15') = 18.64 + 5.30(\pm 2.00) \cdot Sugar\_content \quad \text{(S8)}$$

$n = 12$, $r = 0.642$, $F = 7.0$, $s = 10.1$, $p < 0.025$

$$\bar{v}_1 = 1.14(\pm 0.40) + 0.221(\pm 0.030) \cdot Sugar\_content \quad \text{(S9)}$$

$n = 12$, $r = 0.920$, $F = 54.8$, $s = 0.15$, $p < 0.00002$

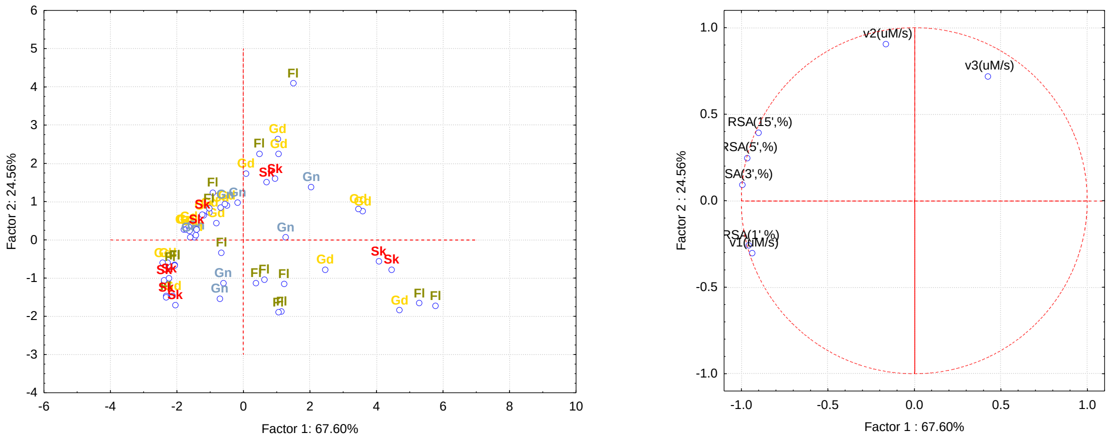

(a) (b)

Figure S10. PCA results for the antioxidant activity and DPPH· kinetics data of all organic and non-organic apple extracts by variety ("Golden Delicious" – "*Gd*", orange, "Florina" – "*Fl*", green, "Generos" – "*Gn*", turquoise, "Starkrimson" – "*Sk*", red): (a) *PC*$_2$ *versus PC*$_1$ scores plot; (b) *PC*$_2$ *versus PC*$_1$ loadings plot.

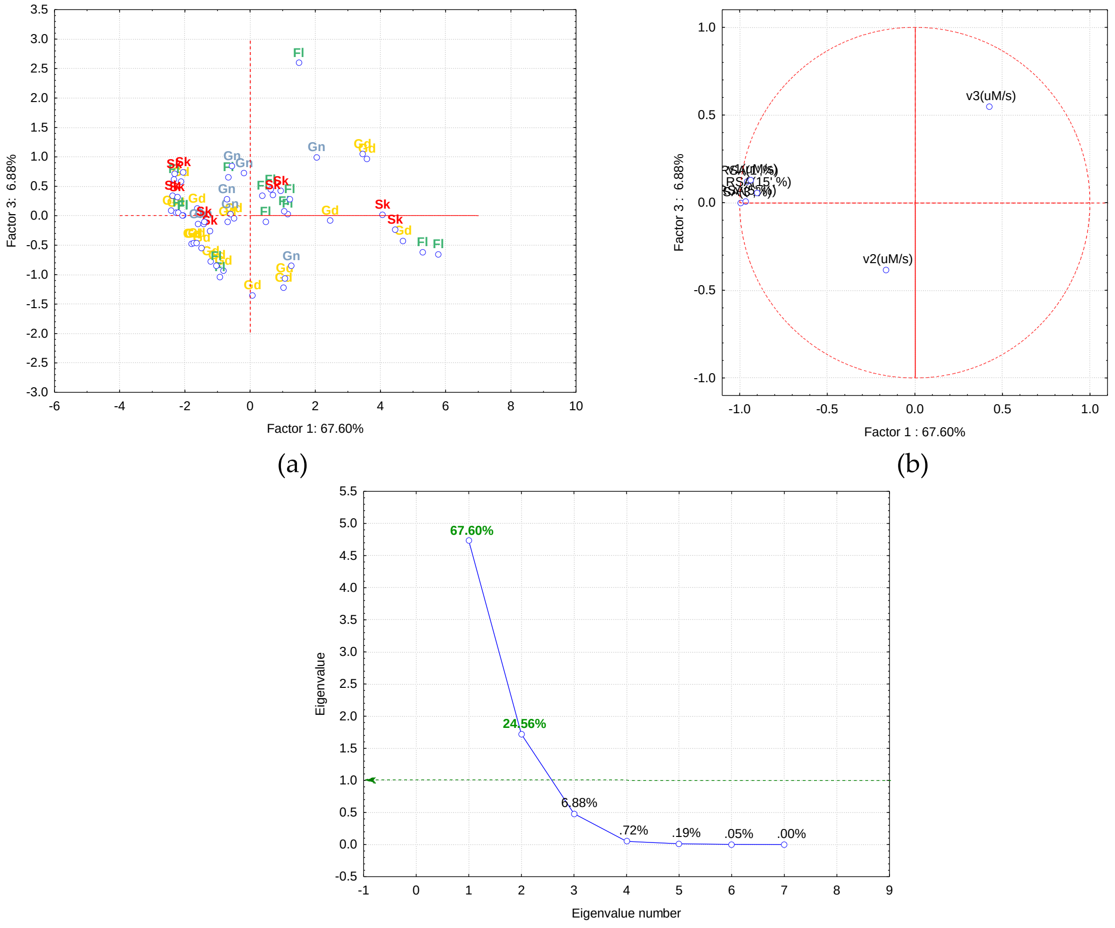

(a) (b)

(c)

Figure S11. PCA results for the antioxidant activity and DPPH· kinetics data of all organic and non-organic apple extracts by variety ("Golden Delicious" – "*Gd*", orange, "Florina" – "*Fl*", green, "Generos" – "*Gn*", turquoise, "Starkrimson" – "*Sk*", red): (a) *PC*$_3$ *versus PC*$_1$ scores plot; (b) *PC*$_3$ *versus PC*$_1$ loadings plot; (c) eigenvalues of the correlation matrix.

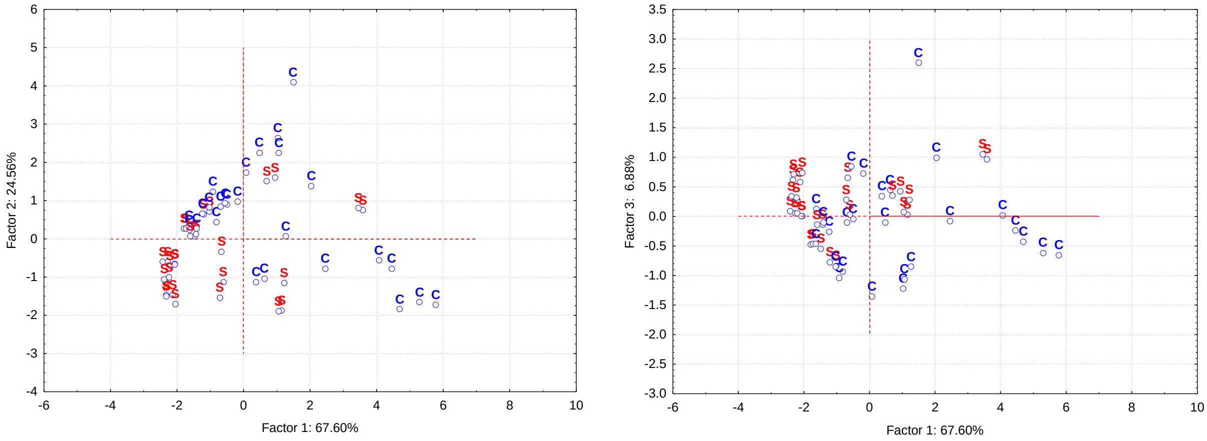

(a) (b)

Figure S12. PCA results for the antioxidant activity and DPPH· kinetics data of all organic and non-organic apple extracts by the fruit part (shell – "*S*", red, and core – "*C*", blue): (a) *PC₂ versus PC₁* scores plot; (b) *PC₃ versus PC₁* scores plot.

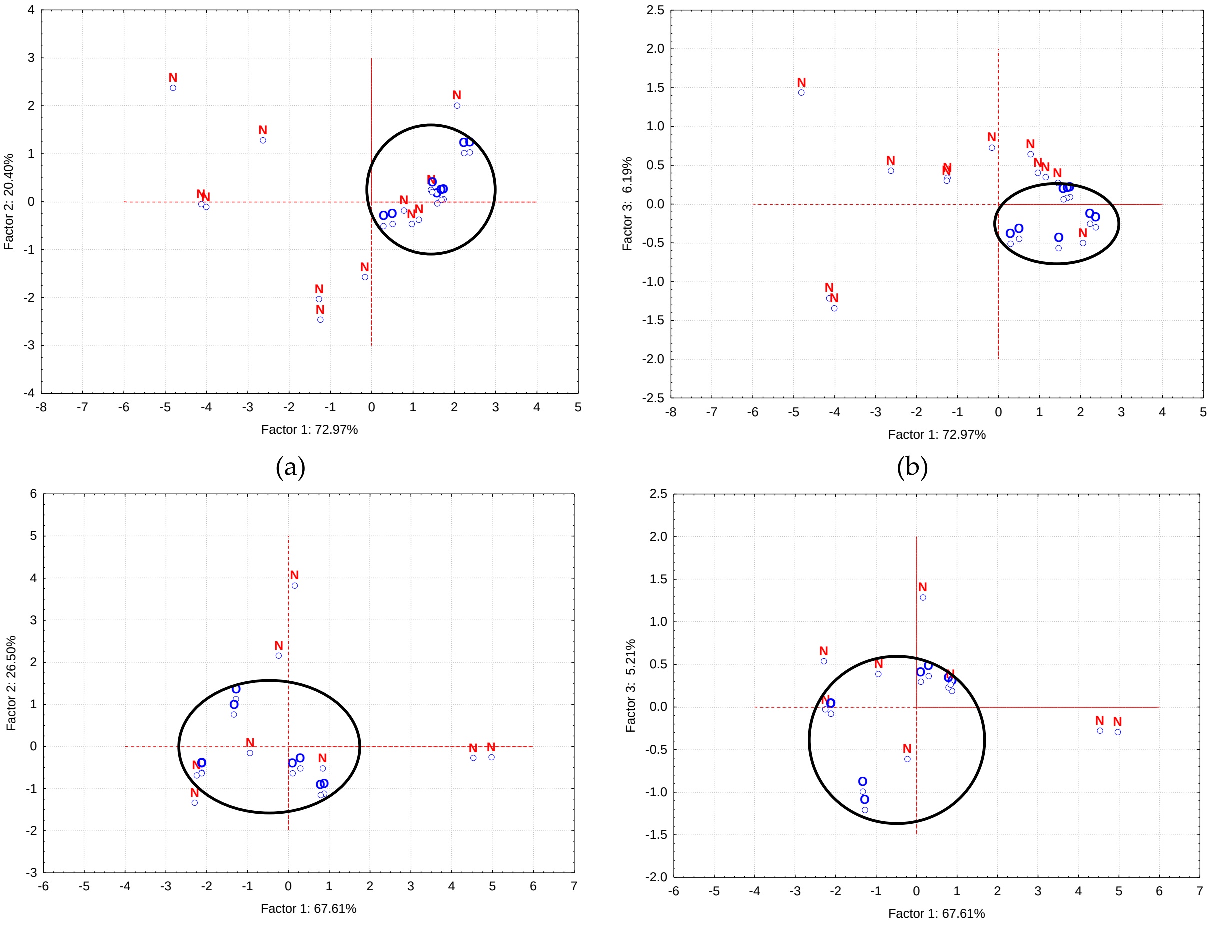

(c) (d)

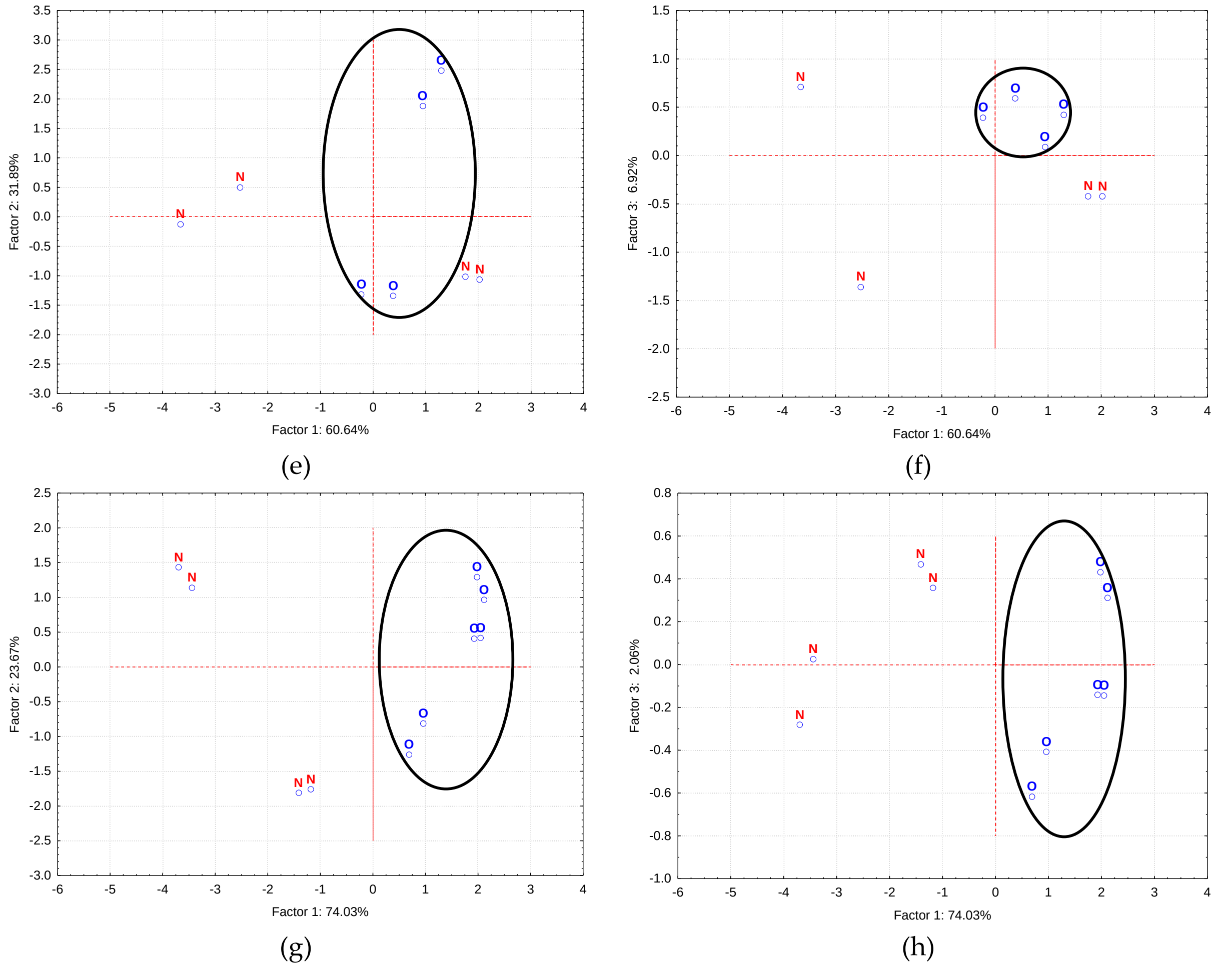

Figure S13. PCA results for the antioxidant activity and DPPH· kinetics data of organic ("*O*" - blue) and non-organic ("*N*" - red) apple extracts: (a) *PC₂ versus PC₁* scores plot for "Golden Delicious" apple variety; (b) (a) *PC₃ versus PC₁* scores plot for "Golden Delicious" apple variety; (c) *PC₂ versus PC₁* scores plot for "Florina" apple variety; (d) *PC₃ versus PC₁* scores plot for "Florina" apple variety; (e) *PC₂ versus PC₁* scores plot for "Generos" apple variety; (f) *PC₃ versus PC₁* scores plot for "Generos" apple variety; (g) *PC₂ versus PC₁* scores plot for "Starkrimson" apple variety; (h) *PC₃ versus PC₁* scores plot for "Starkrimson" apple variety.

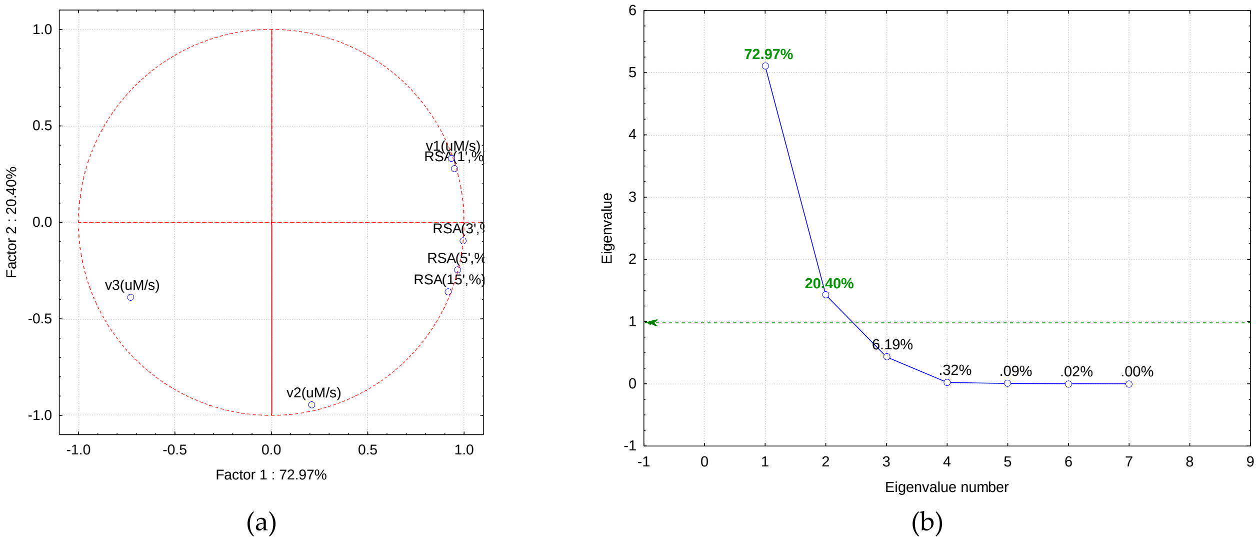

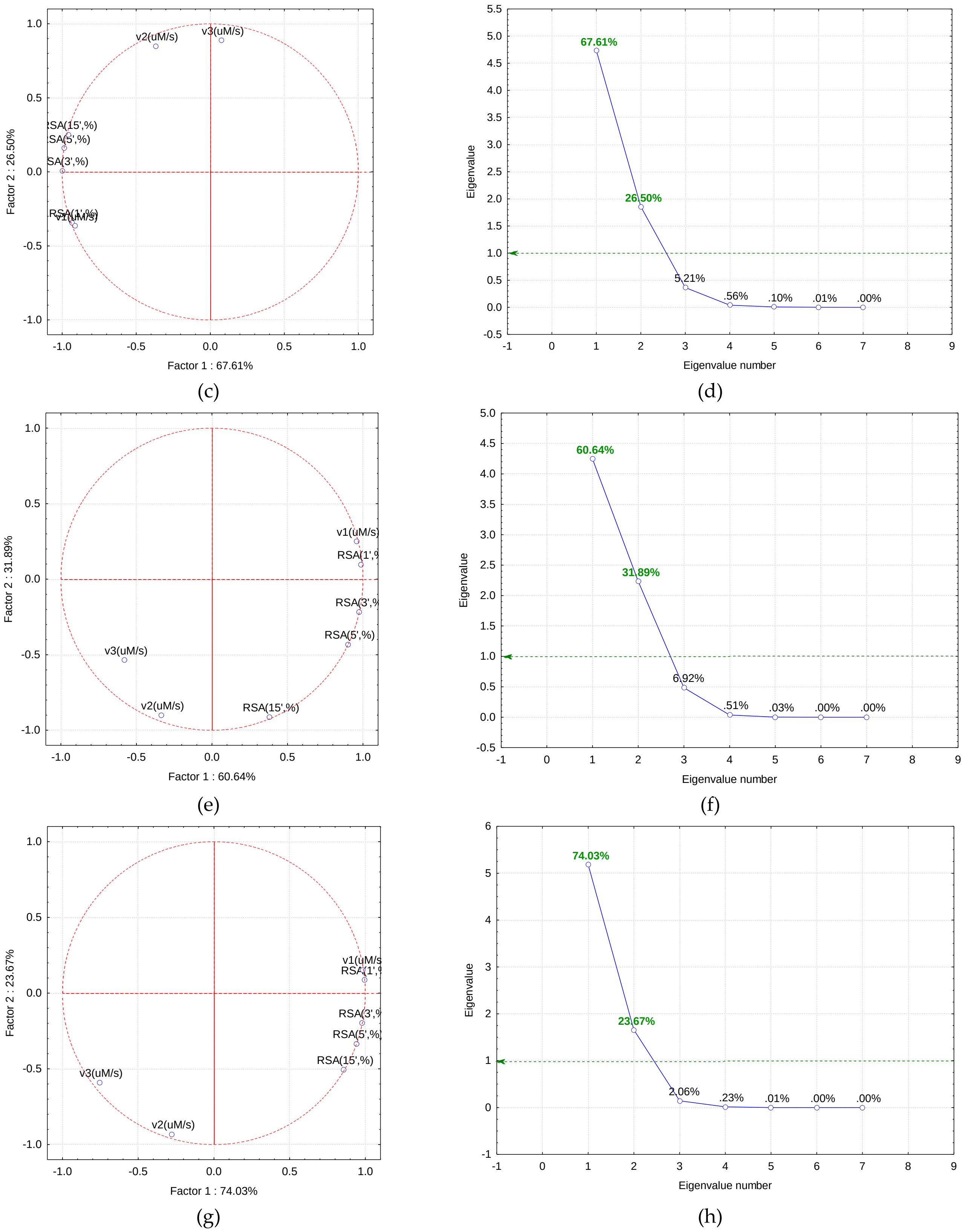

Figure S14. PCA results for the antioxidant activity and DPPH· kinetics data of organic and non-organic apple extracts: (a) PC_2 *versus* PC_1 loadings plot for "Golden Delicious" apple variety; (b) eigenvalues of the correlation matrix for "Golden Delicious" apple variety; (c) PC_2 *versus* PC_1 loadings plot for "Florina" apple variety; (d) eigenvalues of the correlation matrix for "Florina" apple variety; (e) PC_2 *versus* PC_1 loadings plot for "Generos" apple variety; (f) eigenvalues of the correlation matrix for "Generos" apple variety; (g) PC_2 *versus* PC_1 loadings plot for "Starkrimson" apple variety; (h) eigenvalues of the correlation matrix for "Starkrimson" apple variety.
